# Engineering and Two-Stage Evolution of a Lignocellulosic Hydrolysate-Tolerant *Saccharomyces cerevisiae* Strain for Anaerobic Fermentation of Xylose from AFEX Pretreated Corn Stover

**DOI:** 10.1371/journal.pone.0107499

**Published:** 2014-09-15

**Authors:** Lucas S. Parreiras, Rebecca J. Breuer, Ragothaman Avanasi Narasimhan, Alan J. Higbee, Alex La Reau, Mary Tremaine, Li Qin, Laura B. Willis, Benjamin D. Bice, Brandi L. Bonfert, Rebeca C. Pinhancos, Allison J. Balloon, Nirmal Uppugundla, Tongjun Liu, Chenlin Li, Deepti Tanjore, Irene M. Ong, Haibo Li, Edward L. Pohlmann, Jose Serate, Sydnor T. Withers, Blake A. Simmons, David B. Hodge, Michael S. Westphall, Joshua J. Coon, Bruce E. Dale, Venkatesh Balan, David H. Keating, Yaoping Zhang, Robert Landick, Audrey P. Gasch, Trey K. Sato

**Affiliations:** 1 DOE Great Lakes Bioenergy Research Center, University of Wisconsin-Madison, Madison, Wisconsin, United States of America; 2 Department of Chemistry, University of Wisconsin-Madison, Madison, Wisconsin, United States of America; 3 Department of Bacteriology, University of Wisconsin-Madison, Madison, Wisconsin, United States of America; 4 DOE Great Lakes Bioenergy Research Center, Michigan State University, East Lansing, Michigan, United States of America; 5 Biomass Conversion Research Laboratory, Department of Chemical Engineering and Materials Science, Michigan State University, Lansing, Michigan, United States of America; 6 School of Food and Bioengineering, Qilu University of Technology, Jinan, China; 7 Advanced Biofuels Process Demonstration Unit, Lawrence Berkeley National Laboratory, Emeryville, California, United States of America; 8 Deconstruction Division, Joint BioEnergy Institute, Emeryville, California, United States of America; 9 Department of Chemical Engineering & Materials Science, Michigan State University, East Lansing, Michigan, United States of America; 10 Department of Biosystems & Agricultural Engineering, Michigan State University, East Lansing Michigan, United States of America; 11 Division of Sustainable Process Engineering, Luleå University of Technology, Luleå, Sweden; 12 Department of Biochemistry, University of Wisconsin-Madison, Madison, Wisconsin, United States of America; 13 Laboratory of Genetics, University of Wisconsin-Madison, Madison, Wisconsin, United States of America; Virginia Tech, United States of America

## Abstract

The inability of the yeast *Saccharomyces cerevisiae* to ferment xylose effectively under anaerobic conditions is a major barrier to economical production of lignocellulosic biofuels. Although genetic approaches have enabled engineering of *S. cerevisiae* to convert xylose efficiently into ethanol in defined lab medium, few strains are able to ferment xylose from lignocellulosic hydrolysates in the absence of oxygen. This limited xylose conversion is believed to result from small molecules generated during biomass pretreatment and hydrolysis, which induce cellular stress and impair metabolism. Here, we describe the development of a xylose-fermenting *S. cerevisiae* strain with tolerance to a range of pretreated and hydrolyzed lignocellulose, including Ammonia Fiber Expansion (AFEX)-pretreated corn stover hydrolysate (ACSH). We genetically engineered a hydrolysate-resistant yeast strain with bacterial xylose isomerase and then applied two separate stages of aerobic and anaerobic directed evolution. The emergent *S. cerevisiae* strain rapidly converted xylose from lab medium and ACSH to ethanol under strict anaerobic conditions. Metabolomic, genetic and biochemical analyses suggested that a missense mutation in *GRE3*, which was acquired during the anaerobic evolution, contributed toward improved xylose conversion by reducing intracellular production of xylitol, an inhibitor of xylose isomerase. These results validate our combinatorial approach, which utilized phenotypic strain selection, rational engineering and directed evolution for the generation of a robust *S. cerevisiae* strain with the ability to ferment xylose anaerobically from ACSH.

## Introduction

As the world’s human population increases, so does the demand for energy. Renewable biofuels offer a route to replace a portion of the finite amounts of liquid petroleum and natural gas-based fuels. Although bioethanol produced from grain has been employed as a partial replacement for gasoline, this process is generally viewed as unsustainable [1]. An alternative to grain-based ethanol, which has been traditionally produced by microbial fermentation of starch sugars, is bioethanol generated from lignocellulosic (LC) sugars derived from renewable and sustainable plant feedstocks [2]. Despite well over two decades of research, microbial-based production of cellulosic ethanol at an industrial scale remains largely unpracticed throughout the world. Part of the reason for this is a number of molecular barriers that have profound impact on the metabolic catabolism of LC sugars, thereby limiting their efficient and cost-effective conversion into ethanol. In particular, the yeast *Saccharomyces cerevisiae*, in its native form, does not convert most LC sugars into ethanol due to insufficient biochemical activities and metabolic inhibition.

Although *S. cerevisiae* excels at fermenting glucose from cornstarch and sugar cane juice, the fermentation of pentose sugars from the hemicellulose component of lignocellulosic biomass is challenging. In particular, xylose, a pentose sugar and a major component of hemicellulose, composes 30–40% of total cell-wall carbohydrate in grasses and some woody biomass [3]. Conversion of xylose to ethanol is crucial to maximize the economic return from fuel production in excess of feedstock and production costs. However, native *S. cerevisiae* cannot efficiently ferment xylose, as most strains have either lost or downregulated the activities of xylose catabolism enzymes [4] and lack specific xylose transporters [5]. To overcome this deficiency, yeast have been engineered to express a minimal enzyme set from native xylose-metabolizing organisms that allow conversion of xylose into xylulose-5-phosphate (X5P), which can then be catabolized by the pentose phosphate pathway into ethanol. Specifically, engineering of *S. cerevisiae* to express xylose reductase (XR) and xylitol dehydrogenase (XDH), or xylose isomerase (XI) alone, has permitted the limited conversion of xylose into xylulose, which can then be phosphorylated to X5P by overexpression of native or exogenous xylulokinase (XK) (for reviews, see [6]–[8]). With additional rational engineering approaches, yeast strains with improved xylose fermentation in lab medium have been created (reviewed in [9]–[11]). Some of these approaches have been employed with varying degrees of success, including metabolic reengineering of *S. cerevisiae* strains through overexpression of native pentose phosphate pathway enzymes [12], [13], deletion of genes such as *PHO13* to improve xylose metabolism [14], and heterologous expression of putative xylose transporters [5], [15]. Experimental directed evolution is another well-utilized means to improve desired phenotypic traits (reviewed in [16]). A combination of rational engineering followed by directed evolution on xylose-containing medium under aerobic [17], [18] or oxygen-limited [10], [12] conditions has generated yeast strains with increased anaerobic xylose consumption rates relative to their parental strains. Most recently, two sequential anaerobic selections of an XR-XDH engineered *S. cerevisiae* strain on xylose resulted in an evolved isolate with a significantly faster anaerobic consumption rate of xylose than its ancestor, although most of the xylose appears to be converted to xylitol and glycerol [19]. These approaches have allowed for effective consumption of xylose in innocuous and nutrient-rich lab medium; however, conversion of complex, LC-derived xylose from lignocellulosic hydrolysates into biofuels is much more challenging.

Before being deconstructed into fermentable sugars, plant biomass requires chemical, thermal and/or mechanical pretreatments that alter cellulose, hemicellulose and lignin organization, thereby allowing hydrolytic enzymes greater access to sugar polymers for faster rates of enzymatic hydrolysis. Numerous pretreatment methods have been developed and they include the use of dilute acid, bases and ionic liquids (IL) (reviewed in [20]). Although it significantly increases the rate and effectiveness of biomass deconstruction, lignocellulose pretreatment generates a number of common degradation products released from plant cell walls. These chemical compounds include hemicellulose-derived acetate and lignin-derived aromatic aldehydes that induce microbial stress by draining reducing cofactors, limiting ATP generation and causing cellular damage (reviewed in [21] and [22]). Additionally, each pretreatment process can generate its own set of dominant stress-inducing compounds, such as furans generated from dilute acid pretreatment [23], [24] and hydroxycinnamic acids in alkaline hydrogen peroxide (AHP) pretreatment [25]. In some cases, the pretreatment compound itself can be a major biological inhibitor, as is the case for the IL, 1-ethyl-3-methylimidazolium acetate ([C2mim][OAc]) [26]. Ammonia Fiber Expansion (AFEX) is a highly effective pretreatment for herbaceous biomass. In contrast to dilute acid, which degrades the hemicellulose, AFEX pretreatment retains hemicellulose as intact polymers [27] that can then be hydrolyzed into fermentable sugars for additional fuel production. However, AFEX pretreatment of corn stover generates diverse inhibitory small molecules, including phenolic amides, which impair xylose fermentation by *S. cerevisiae* [28], [29]. These effects are often compounded during xylose fermentation in the absence of oxygen, likely due to reduced ATP yield from pentose sugars compared to hexoses, combined with decreased energetic yield under anaerobic conditions. Given that ATP drives numerous detoxification processes [21], the cellular stress induced by these compounds has profound impacts on xylose fermentation. To cope with these diverse inhibitory compounds present in LC hydrolysates and their impacts on cellular physiology and metabolism, many researchers have opted to employ industrial or environmental *S. cerevisiae* strains with innate stress tolerant properties [12], [25], [30]–[32]. A combination of directed engineering and evolution approaches with *S. cerevisiae* for xylose metabolism in the presence of defined spruce [30] or AHP [25] hydrolysate inhibitors or undefined raw spruce hydrolysates [12] have resulted in a small number of *S. cerevisiae* strains with improved xylose fermentation properties in lignocellulosic hydrolysates compared to their parental strains.

At present, little is known about how the inhibitors found in AFEX-pretreated corn stover hydrolysate (ACSH) impact xylose fermentation by *S. cerevisiae*, particularly under strict, industrially relevant, anaerobic conditions where ethanol production is maximized. As the foundation for investigating this knowledge gap, and the goal of determining what genetic factors are important for improving anaerobic xylose fermentation, we sought to develop and compare closely related *S. cerevisiae* strains with varying anaerobic xylose fermentation phenotypes. Previously, we identified a natural *S. cerevisiae* strain, GLBRCY0 (originally named as NRRL YB-210 [33]) with growth tolerance to individual LC-derived inhibitors [25] as well as ACSH at elevated temperature [34]. Despite engineering NRRL YB-210 (YB-210) with known XR-XDH genes, the strain fermented xylose in lab medium and ACSH at slow rates, even in the presence of limited oxygen. Here, we report the development of an engineered and evolved derivative of YB-210, which displayed robust cell growth in a variety of pretreated lignocellulosic hydrolysates (LCH) relative to other strains, with the ability to rapidly ferment xylose from lab medium and ACSH under completely anaerobic conditions. Combined genetic and metabolomic analyses indicate that the evolved strain, named GLBRCY128 (Y128), incurred a missense mutation in *GRE3*, which, combined with additional unknown mutations, allowed for faster anaerobic xylose consumption rates relative to its parent. Together, these results identify Y128 as a novel *S. cerevisiae* strain with evolved genetic traits for robust anaerobic fermentation of xylose from ACSH. They also illustrate how careful selection of genetic background can accelerate development of biocatalysts with the ability to ferment xylose anaerobically in an inhibitor-laden LCH.

## Materials and Methods

### AFEX pretreated corn stover hydrolysate (ACSH) preparation

*Zea mays* (Pioneer hybrid 36H56) corn stover from Field 570-C Arlington Research Station, University of Wisconsin was harvested in 2008 for use in 96-well plate phenotyping or in 2009 for use in anaerobic fermentation experiments. AFEX pretreatment of corn stover was performed as described previously [27]. AFEX pretreated corn stover was hydrolyzed at 6% or 9% glucan loading in a 1.5 L reaction volume in a 3 L Applikon fermenter (Applikon Biotechnology Inc. USA) with Spezyme CP (15 FPU/g glucan loading, DuPont Danisco), Multifect Xylanase (10% of Spezyme CP, DuPont Danisco), and Novovzyme 188 (64 pNPGU/g glucan, Sigma-Aldrich) at 50°C for 5 days. Tetracycline (40 mg/L) was used to prevent microbial contamination and pH 4.8 was maintained during the hydrolysis process. Biomass was added to the reaction mixture in 4 batches within 4 h from the start of hydrolysis to facilitate better mixing at 1000 rpm. After 120 h, the hydrolysis mixture was centrifuged (2500×g for 30 min.) and sterile filtered (0.22 µm pore size; Millipore Stericup). For 6% glucan loading ACSH, the final sugar concentrations were 53 g/L glucose and 21.7 g/L xylose. For 9% glucan loading ACSH, the final sugar concentrations were 80 g/L glucose and 36 g/L xylose. For ACSH used in bioreactor fermentations, hydrolysates were prepared as described previously [35] with one additional modification; prior to fermentation, the hydrolysate was adjusted to pH 5.0 and again filtered through a 0.2 µm filter to remove precipitates and to ensure sterility.

### Alkaline hydrogen peroxide pretreated hydrolysates

Pioneer hybrid 36H56 corn stover described above and switchgrass (*Panicum virgatum* cv. Cave-In-Rock) described elsewhere [36] were milled (Circ-U-Flow model 18-7-300, Schutte-Buffalo Hammermill, LLC) to pass through a 5 mm screen. AHP pretreatment was performed as reported previously [37] at a hydrogen peroxide loading of 0.125 g H_2_O_2_/g biomass in an incubator with shaking at 150 rpm at 30°C for 24 h with periodic pH readjustment to 11.5 during pretreatment using 5 N NaOH. For switchgrass, pretreatment was conducted at biomass concentration of 20% w/w. For corn stover, pretreatment was conducted at biomass concentration of 15% w/w. Following pretreatment, the whole slurry was adjusted to pH 4.8 using 72% H_2_SO_4_. Accelerase 1000 (Novozymes A/S), Multifect xylanase, and Multifect pectinase (DuPont Danisco) were used at the protein ratio of 0.62∶0.24∶0.14 with a total protein loading of 30 mg/g glucan as assayed by the Bradford Assay. Hydrolysis was performed at 50°C with shaking speed of 180 rpm for 24 h. After enzymatic hydrolysis, the whole slurry was centrifuged at 18,000×g for 30 min. The supernatant was used as undetoxified raw hydrolysate or for detoxified hydrolysate, activated carbon (Fisher Scientific #05-690A) was mixed with undetoxified hydrolysate at 5% concentration (5 g activated carbon with 100 mL hydrolysate) and incubated at 50°C for 1 h in an unbaffled shake flask at 150 rpm. After centrifugation at 18000×g for 30 min, the supernatant was used as the detoxified hydrolysate. All hydrolysates were filter-sterilized (0.22 µm pore size; Millipore Stericup). Final sugar concentrations for AHP hydrolysates were 30 g/L glucose and 20 g/L xylose for raw AHP corn stover hydrolysate, 35 g/L glucose and 23 g/L xylose for detoxified AHP CSH, and 27 g/L glucose and 19 g/L xylose for both raw and detoxified AHP SGH.

### Dilute acid pretreated lignocellulosic hydrolysates

An industrial collaborator provided two different versions of dilute acid pretreated wheat straw that was hydrolyzed using a proprietary blend of cellulase enzymes at pH 5.0 and 50°C. Both hydrolysates were diluted 4∶5 in sterile water supplemented with 10 g/L yeast extract and 20 g/L peptone.

### Ionic liquid pretreated switchgrass hydrolysate (IL-SGH)

Switchgrass was pretreated with [C2mim][OAc] (1-ethyl-3-methylimidazolium acetate) at 15% solids loading as described elsewhere [38]. IL-pretreated switchgrass was hydrolyzed with CTec2 (54 mg/g glucan) and HTec2 (6 mg/g glucan) enzymes (Novozyme) for 72 h in a 2 L IKA bioreactor. [C2mim][OAc]-pretreated SGH was generated at Advanced Biofuels Process Demonstration Unit (batch ABPDU 110201S02). Final sugar concentrations in the IL-SGH were 41 g/L glucose and 10 g/L xylose.

### Lab media

Standard undefined yeast lab medium was prepared as previous described [39]. Briefly, liquid and plate-based medium contained 10 g/L yeast extract and 20 g/L peptone (YP), and various sugar concentrations (X = 20 g/L xylose, D = 20 g/L dextrose or glucose, DX = 60 g/L glucose and 30 g/L xylose). Where indicated, hydrolysates were supplemented with 10 g/L yeast extract and 20 g/L peptone. For bioreactor experiments, this YPDX medium also contained 50 mM potassium phosphate, pH 5.0.

### *Saccharomyces cerevisiae* strains, 96-well growth assays and hierarchical clustering

Native *S. cerevisiae* strains used in this study (see **Table S1**) were obtained from Dr. Cletus Kurtzmann (USDA ARS, Peoria, IL), National Collection of Yeast Cultures (Norwich, UK), and Dr. Justin Fay (Washington University, Saint Louis, MO). Aerobic growth assays in microtiter plates were performed as previously described [40] [25] [34], with one exception; 10 µL of saturated culture was inoculated into 190 µL of YPD or a single type of pretreated lignocellulosic hydrolysate. Cell growth was measured by optical density at 595 nm every 10 min for 24 h in Tecan F500 or M1000 multimode plate readers with an interior temperature of 30°C. Background-subtracted cell density readings for each strain were analyzed by the program GCAT [25]. Normalized specific growth rates for each strain from three independent biological replicates in pretreated hydrolysates were normalized to their average growth rate in YPD alone, and then ranked ordered from 1 to 117 (including control strains – Y389, BY4741, CEN.PK113-5D and CEN.PK2-1D in duplicate) for highest average specific growth to lowest, respectively. For all strains with no detectable specific growth rates, strains were assigned a rank of 117. Strain ranks in each medium condition were hierarchically clustered and displayed with Spotfire (TIBCO).

### DNA constructs and strain engineering

Genotypes of engineered strains used in this study are described in **Table 1**. Construction of the GLBRCY73 strain has been described elsewhere [40]. The expression cassette containing *TAL1* from *S. cerevisiae* S288c (*ScTAL1*), *xylA* from *Clostridium phytofermentans* ISDg (*CpxylA*) and *XYL3* from *Scheffersomyces stipitis* CBS 6054 (*SsXYL3*) was generated in a similar manner with some modifications. Codon-optimized versions of each gene were synthesized (GeneArt, Life Technologies) and inserted via homologous recombination in the following promoter-open reading frame-terminator combinations in order from 5′ to 3′: *ScPGK1* promoter-*ScTAL1*-*ScTDH3* terminator, *ScTDH3* promoter-*CpxylA*-*ScTEF2* terminator, *ScTEF2* promoter-*SsXYL3*-*ScCYC1* terminator. This cassette, which also contains a loxP-KanMX-loxP selection marker [41], was flanked by *ScHO* sequences [42] for targeted recombination at the genomic *HO* locus. The complete *CpxylA* cassette was amplified by standard polymerase chain reaction (PCR) using primers that anneal to the 5′ ends of the *HO* flanking sequences, gel purified and transformed into the NRRL YB-210 strain. Following selection on YPD plates containing 200 µg/mL Geneticin (Life Technologies), verification of cassette insertion was determined by PCR using combinations of primers that anneal outside of the HO flanking sequence and specific to synthesized DNA cassette. The engineered YB-210 diploid strain was then subjected to sporulation and tetrad dissection. One spore, which was derived from a tetrad with three other inviable spores, was confirmed for a single MATa mating type and subsequently named GLBRCY22-3 (Y22-3). LoxP-KanMX-loxP marker rescues from Y22-3, Y127 and Y128 were carried out by expression of Cre recombinase as published elsewhere [41] to generate the respective Y36, Y132 and Y133 strains. Deletion of engineered *CpxylA* and *ScTAL1*, and endogenous *ScGRE3* were performed by integration of PCR product using a loxP-KanMX-loxP cassette [41] into the marker-rescued versions of GLBRCY36, GLBRCY132 and GLBRCY133 strains. Sanger sequencing of PCR products and DNA plasmids was performed by the University of Wisconsin-Madison Biotechnology Center.

**Table 1:** Engineered and evolved *S. cerevisiae* strains used in this study.

Strain name	Genotype	Reference
GLBRCY73	NRRL YB-210 MATa/α *HOΔ::SsXYL1-SsXYL2-SsXYL3-loxP-KanMX-loxP*, aerobically evolved on YPX	[40]
GLBRCY22-3	NRRL YB-210 MATa spore *HOΔ::ScTAL1-CpxylA-SsXYL3-loxP-KanMX-loxP*	This study
GLBRCY127	GLBRCY22-3 MATa, aerobically evolved isolate on YPDX	This study
GLBRCY128	GLBRCY127 MATa, anaerobically evolved isolate on YPDX	This study
GLBRCY36	GLBRCY22-3 with *loxP-KanMX-loxP* marker excised by Cre	This study
GLBRCY132	GLBRCY127 with *loxP-KanMX-loxP* marker excised by Cre	This study
GLBRCY133	GLBRCY128 with *loxP-KanMX-loxP* marker excised by Cre	This study
GLBRCY132 *xylAΔ*	GLBRCY132 *xylAΔ::loxP-KanMX-loxP*	This study
GLBRCY132 *tal1Δ*	GLBRCY132 synthetic *tal1Δ::loxP-KanMX-loxP*	This study
GLBRCY133 *xylAΔ-A*	GLBRCY133 *xylAΔ::loxP-KanMX-loxP* transformant A	This study
GLBRCY133 *xylAΔ-B*	GLBRCY133 *xylAΔ::loxP-KanMX-loxP* transformant B	This study
GLBRCY36 *gre3Δ*	GLBRCY36 *gre3Δ::loxP-KanMX-loxP*	This study
GLBRCY132 *gre3Δ*	GLBRCY132 *gre3Δ::loxP-KanMX-loxP*	This study
GLBRCY133 *gre3Δ*	GLBRCY132 *gre3Δ::loxP-KanMX-loxP*	This study

### Quantitative RT-PCR

GLBRCY128 and GLBRCY128 *xylAΔ* strains were aerobically cultured at 30°C in baffled shake flasks containing YPD media. When the culture reached log phase growth (OD_600_ = 0.8), 7 mL of cell culture was then harvested by centrifugation at 3,000×*g* for 3 min, the supernatant was decanted and then cell pellets were flash frozen on dry ice/ethanol. Frozen cell pellets were resuspended and then vortexed in 0.8 mL phenol, pH 4.3 and 0.8 mL lysis buffer (10 mM Tris-Cl, pH 7.4, 10 mM EDTA, 0.5% SDS). Cell lysates were incubated at 65°C for 30 min and then centrifuged at 20,000×*g* at 4°C. The aqueous phase was then transferred to a new 2 mL centrifuge tube and further extracted with two additional rounds of phenol and then chloroform. The final extracted aqueous phase was then transferred to RNase-free minicentrifuge tubes and RNA precipitated by addition of 0.1 volumes of 0.3 M NaOAc, pH 5.2 and 2.5 volumes of 100% ethanol at −20°C for 1 h. Precipitated RNA was pelleted by centrifugation at 20,000×*g* for 30 min at 4°C. The RNA pellet was washed with 2 mL 70% ethanol, dried in a Speed-Vac, and dissolved in 0.2 mL RNase-free TE buffer (10 mM Tris-HCl, 1 mM EDTA, pH 8.0). RNA was further purified by RNeasy Mini Kit (Qiagen) according to the manufacturer protocol. cDNA synthesis from 10 µg total RNA was performed with Superscript III reverse transcriptase (Life Technologies) according to manufacturer protocol. Generated cDNA was purified and concentrated with a PCR Minelute Purification kit (Qiagen) into 12 ml elution buffer according to manufacturer protocol. For quantitative reverse-transcriptase polymerase chain reaction (qPCR) of *xylA*, 10 ng of cDNA was mixed with 500 nM SynCpXylA FWD (5′-GGTGGATGCTAGGTTGTCTTT-3′) and 250 nM SynCpXylA REV (5′-CACGCCTTCTTGCTCAAATAAC-3′), or ScERV25qPCRfor and ScERV25 FWD (5′-GTCGCGGATATTCACTCAGATG-3′) and ScERV25 REV (5′-CCTGCAAAGTCCCTCTTTCTAC-3′) primers and SYBR JumpStart Taq Ready Mix with Rox internal standard (Sigma-Aldrich) according to manufacturer protocol. Relative quantities of *CpxylA* and *ScERV25* RNA were determined using an ABI7500 Real-Time PCR instrument (Applied Biosystems). Relative concentrations of *CpxylA* and *ScERV25* transcripts were determined by ΔC_t_ method.

### Directed Evolution

Cell density measurements were determined from OD_600_ measurements from the cultures diluted 1∶10 in 1 cm path length cuvettes by a Beckman Coulter DU720 spectrophotometer. For aerobic adaptation, GLBRCY22-3 was inoculated at OD_600_ = 0.05–0.1 in 250 mL YP medium containing 2% xylose and 0.1% glucose. The first 15 transfers took place over 53 days with serial 1∶10 dilutions in fresh medium occurring every 3–4 days. After transfer 15, the adaptation culture was diluted every 2–3 days over the course of 44 additional days, ending after transfer 34. For anaerobic adaptation, GLBRCY127 was inoculated at OD_600_ = 0.05–0.1 in a flask containing 50 mL YP medium with 2% xylose, 0.1% glucose and 50 µg/mL Geneticin, and then placed in an anaerobic chamber. For the first two anaerobic transfers, the medium also contained 40 µg/L ergosterol (Sigma-Aldrich) and 4 g/L Tween-80 (Sigma-Aldrich). Anaerobic cultures were maintained in suspension using a stir bar and magnetic stir plate, and passaged every 7 days during the first 5 transfers. After the 5^th^ transfer, the culture was passaged every 3–4 days with the final 14^th^ transfer finishing 66 days after the start of the anaerobic adaptation. Xylose concentrations in the medium at the end of each transfer cycle were measure by YSI 2700 Select instrument. At the end of each adaptation, the culture at 1∶10,000 dilution was spread onto multiple YPD-Geneticin plates and incubated at 30°C for 48 h. Single clonal isolates were picked and evaluated for growth in YPX medium either aerobically or anaerobically in an anaerobic chamber as described previously [25], [40].

### Aerobic and anaerobic fermentations

Aerobic and anaerobic batch fermentations in 3 L bioreactors (Applikon Biotechnology) were conducted using 2.1 L of ACSH or 1.7 L of YPX or YPDX with 50 mM potassium phosphate medium. Vessels were sparged in the headspace with N_2_ (150 sccm) for anaerobic fermentation or in the medium with air (200 sccm) for aerobic fermentation. Inocula were prepared from single colonies grown in YPD-Geneticin medium aerobically for ∼9 h, and then were diluted in ACSH or YPD medium with Geneticin at an initial OD_600_ = 0.1 (approximately 0.08–0.1 g DCW/L), and then grown aerobically for approximately 20 h. Starter cultures were then inoculated at a starting OD_600_ = 0.1 in bioreactor vessels maintained at 30°C and pH 5.0 with NaOH or HCl, and stirred at 500 rpm. For aerobic and anaerobic YPX growth assays, inoculum cultures were started from single colonies grown in YPD-Geneticin medium overnight and then diluted to OD_600_ = 0.1 in YPX (no Tween-80 or ergosterol) at the start of the assay. Yeast cultures were grown in culture tubes containing 5–7.5 mL of medium shaken at 30°C, or in 30 mL of medium stirred in flasks placed in an anaerobic chamber (Coy) purged with hydrogen. For anaerobic experiments, bioreactors containing YPX, YPDX or ACSH were sparged with N_2_ into the medium for at least 2 h. Flasks containing YPX were placed in the anaerobic chamber for at least 2 h prior to inoculation. Cell density measurements were determined by OD_600_ measurements from cultures diluted 1∶10 or 1∶25 in water. All OD_600_ measurements were blanked against uninoculated medium diluted in the same manner. Dry cell weight (DCW) was determined by vacuum filtering 50 mL of culture at 4 time points onto pre-weighed filters, washing with water and microwaving on 10% power for 15 minutes. Filtered cells were additionally dried by desiccant for 2–3 days and then weighed. DCW values in grams per 50 mL of culture included subtraction of the filter weight alone. Correlations between g DCW/L and OD_600_ concentrations were calculated to provide cell density measurements based on cell mass/L. Medium glucose and xylose concentrations from aerobic tube and anaerobic flask experiments were determined by YSI instrument. Extracellular glucose, xylose, ethanol, glycerol and xylitol concentrations from aerobic and anaerobic bioreactor experiments were determined by high performance liquid chromatography (HPLC) and refractive index detection (RID) as published elsewhere [35].

### Quantification of intracellular pentose metabolic intermediates

To quantify intracellular metabolites, 5–10 mL of cell culture was rapidly removed from bioreactors with a 20 mL sterile syringe and 4 mL aliquots were applied to a filtration manifold unit (Hoefer FH 225 V) outfitted with sterile 25 mm nylon filters (Whatman; Nylon; 0.45 µm pore size), and the cells captured on the filters under vacuum. To reduce the background associated with metabolites present in ACSH, the cells were then rapidly washed with 5 mL of synthetic hydrolysate media [35] at pH 5.0 lacking amino acids and replacing 9% sorbitol in place of 6% glucose and 3% xylose. The filters were then removed and rapidly placed in 15 mL conical tubes containing ice-cold extraction buffer ([43]; acetonitrile-methanol-water, 40∶40∶20, 0.1% formic acid) and flash frozen in a dry ice ethanol bath.

The concentration of xylulose-5-phosphate was determined using reverse phase ion pairing HPLC [44] and electrospray ionization tandem mass spectrometry (ESI-MS/MS). Reagents and nonlabeled reference compounds were from Sigma Aldrich Co. (Saint Louis, Missouri, USA). Compounds were separated on an Ascentis HSS-T3 C18 column, 150×2.0 mm, 1.8 µm particle size (Waters Acquity). The mobile phase A consisted of 92.5∶7.5 water:methanol, 10 mM tributylamine, and 15 mM acetic acid, and mobile phase B was isopropyl alcohol. Xylulose-5-phosphate, whose peak overlapped with ribulose-5-phosphate, was quantified by integrating the portion of the partially resolved peak that was clearly attributable to X5P.

For quantifying intracellular xylose, xylulose, and xylitol, 20 µL aliquots of metabolite extract were transferred to 2.5 mL centrifuge tubes along with 20 µL of a solution containing 100 µM U-^13^C_5_-xylitol (Sigma-Aldrich), 100 µM U-^13^C_5_-xylose (Sigma-Aldrich) and 50 µM U-^13^C_5_ xylulose. U-^13^C_5_ xylulose was obtained by enzymatic conversion of U-^13^C_5_-xylose by immobilized xylose isomerase (Sigma-Aldrich). Samples were then evaporated to dryness under reduced pressure in a rotary evaporator (Savant SPD131A) with cryogenic cold trap for 3–4 hours. Dried samples were incubated with 30 µL 2% methoxyamine hydrochloride in anhydrous pyridine at 60°C for 45 min, and then derivatized at 60°C for an additional 30 min with 70 µL N-methyl,N-(trimethylsilyl) trifluoroacetamide with 1% trimethylchlorosilane (Fisher Scientific). Derivatized samples were then analyzed by gas chromatography coupled with mass spectrometry (GC-MS) on an Agilent 5975 MSD with a Combi PALl autosampler (CTC analytics), and a 6890A GC oven equipped with a 30 m×0.25 mm ID×0.25 µm film HP5-MS capillary column. The inlet was held at 250°C and operated in split mode with a ratio of 10∶1 with a helium carrier gas flow rate maintained at 1.2 mL/min. The oven temperature was held at 125°C for 47 min then increased linearly at 40°C/min to a final temperature of 300°C.

The mass spectrometer was operated in SIM mode divided into time segments so that only the relevant masses were monitored over the times when each target compound eluted, allowing optimal dwell times of 100–150 ms while still recording at least 20 points over the width of a peak. SIM masses were selected corresponding to fragments (M^+^–15) containing all 5 ^13^C atoms for the labeled internal standards of xylose, xylulose (m/z 457) and xylitol (m/z 427) to allow detection without interference from the isotopic masses of the coeluting natural abundance compounds. The naturally-occurring ^12^C compounds were measured by peak areas of the corresponding ^12^C (xylose, m/z 452, xylitol, m/z 422) ions except for xylulose, which was monitored with a much more abundant m/z 263 ion that was found to be free of interference from the labeled internal standard.

Instrument operation, data collection, and calculation of results were conducted by Agilent MassHunter for GC software VB.07.00 and Mass Hunter Workstation Quantitative Analysis v.B.06.00. Results were calculated from relative peak areas of analytes to their corresponding internal standards interpolated with a calibration curve of relative natural standard/^13^C internal standard peak areas versus relative standard/^13^C internal standard concentrations.

### *In vitro* xylose reductase activity assays

*In vitro* xylose reductase (XR) activities were performed as previously described [45]–[47] with minor modifications. Y132, Y132 *gre3Δ*, Y133 and Y133 *gre3Δ* strains were grown in YPD medium to OD600 = 0.8 and then cells from 45 mL of culture were harvested, washed with 10 mL 0.85% NaCl and re-suspended in an equal volume of breaking buffer (50 mM potassium phosphate, pH 7.4, 1 mM β-mercaptoethanol and 1x Thermo HALT protease inhibitor cocktail). Resuspended cells were then transferred to a glass tube with glass beads and vortexed. The resulting material was centrifuged at 4**°**C and the clarified cell lysate used immediately for activity assays. A Tecan M1000 microplate reader was used to measure absorbance at 340 nm in technical triplicates. Protein concentrations from cell extracts were determined using a modified Bradford assay [48] with bovine serum albumin as the standard. Units of enzyme activity were normalized to total protein extract concentration and averaged from two independent biological samples. Specific XR activities were measured in range of 2–40 mU activity/mg total protein in the presence of NADPH co-factor.

## Results and Discussion

### Phenotypic screening identifies natural *S. cerevisiae* strains tolerant to pretreated lignocellulosic hydrolysates

The primary goal of this study was to create a strain of *S. cerevisiae* that can effectively ferment xylose anaerobically from AFEX-pretreated lignocellulosic biomass. We first evaluated two well-characterized laboratory strains, BY4741 [49] and CEN.PK2 [50] as potential starting points of this research by evaluating their growth abilities in lignocellulosic hydrolysates (LCHs) generated from a variety of established pretreatments and feedstocks (see Materials and Methods). Although both strains reached saturated cell density within 8 h after inoculation in YPD medium, they grew at substantially slower rates and reached low cell densities in the pretreated LCHs compared to standard medium (**Figs. S1A** and **B**), even though glucose concentrations were significantly higher in LCHs. These results suggest that the BY4741 and CEN.PK2 lab strains would not be able to generate sufficient cell biomass for rapid fermentation of inhibitor-laden hydrolysates. Thus, we sought an alternative strain background with robust cell growth in multiple LCHs in hopes of utilizing a strain with sufficient tolerance to LCH inhibitors. To find such strain, we performed comprehensive phenotyping of a collection of publicly available wild and domesticated *S. cerevisiae* strains obtained from a variety of locations and environments (Supplemental Strain Table 1, [51]–[53]) for cell growth in multiple pretreated LCHs. Individual strains were inoculated into 96-well plates containing 6% or 9% glucan-loading AFEX pretreated corn stover (ACS), raw or detoxified AHP pretreated corn stover (CS) or switchgrass (SG), [C2mim][OAc]-pretreated SG (IL SGH), or two different proprietary dilute acid pretreated biomass materials, supplemented with yeast extract and peptone (YP). Cell densities were continuously measured for 24–48 h, from which specific growth rates for each strain in every medium condition were calculated and normalized relative to their growth rate in YPD medium.

The collection of strains displayed wide ranges of aerobic growth rates in the various hydrolysate conditions (**Figs. S2–11** and **Table S1**). Supplementation of YP to 6% glucan-loading ACSH (**Figs. S2** and **S6**) improved growth, suggesting that growth defects in hydrolysates were due to lack of specific nutrients or additional nutrients allowed strains to overcome the effect of toxins. Not surprisingly, detoxification of AHP CSH and SGH (**Figs. S4, S5, S7** and **S8**) significantly improved overall rates compared to raw AHP hydrolysates, while growth rates in 9% glucan-loading ACSH, which contained higher concentrations of sugars and inhibitory compounds [34], were lower than in 6% glucan medium (**Figs. S2** and **S3**). In AHP CSH (**Fig. S4**) and the dilute acid hydrolysates (**Figs. S9** and **S10**), many strains did not achieve enough cell growth to calculate exponential growth rates, suggesting that these hydrolysates contained higher inhibitor concentrations, different combinations of inhibitors, or both, compared to the other hydrolysates. Direct growth-rate comparisons between different hydrolysate pretreatments were not made since the hydrolysates were not standardized for glucan-loading or feedstock source (e.g., AHP SGH vs. IL SGH).

**Figure 1:**
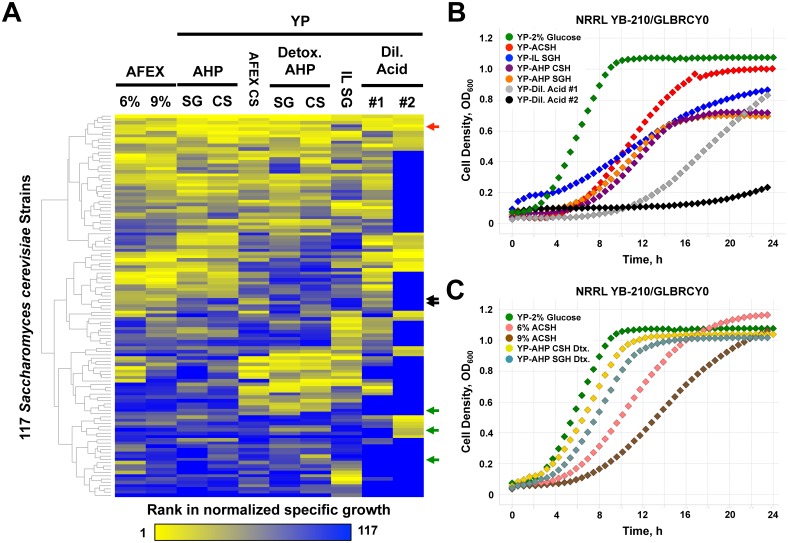
Phenotypic screening of wild and domesticated *S. cerevisiae* strains identifies NRRL YB-210 with tolerance to hydrolysates made from a variety of pretreated lignocellulose. In (**A**), 117 *S. cerevisiae* strains (including some in duplicate) were cultured in 96-well plates and monitored for changes cell density and growth rates calculated as described in Materials and Methods. All strains in each condition were then ranked from 1 (highest growth rate in yellow) to 117 (lowest growth rate, or no growth, in blue) and hierarchically clustered. Arrows indicate clustered rows for BY4741 (green), CEN.PK2 (black) in duplicate microtiter wells, and NRRL YB-210/GLBRCY0 (red). Representative growth data for the YB-210/GLBRCY0 strain in the indicated media from Fig. 2A are plotted (**B–C**). CS, corn stover; SG, switchgrass; YP; Yeast Extract and Peptone supplementation, 6%; 6% glucan loading ACSH, 9%; 9% glucan loading ACSH, Dtx.; Detoxified.

To glean insights from the large datasets we amassed, we used hierarchical clustering to organize strains based on their rank in growth rate for each hydrolysate relative their growth rate rank in YPD medium (the reference media), and according to similarities in phenotypes across strains and conditions (**Fig. 1A**). Clustering the hydrolysates based on the distribution of growth phenotypes showed that AHP and ACS hydrolysates grouped together, regardless of plant feedstock, which is consistent with the fact that these LCHs are generated by alkaline-based pretreatments. AHP and AFEX pretreatments have been shown to produce lower levels of inhibitory furans commonly generated from acid dehydration of hexose sugars, particularly furfural, and this absence may be a significant driver in the clustering of alkaline hydrolysates from acid hydrolysates [23]–[25], [29]. In addition, growth in IL and dilute acid-pretreated LCHs differed significantly from growth in other hydrolysates, with most strains unable to grow in dilute acid pretreated LCH #2. One study suggested that the predominant inhibitor in IL-derived LCHs is residual IL itself [26], which likely drives the unique phenotypic profile of yeast strain growth. It is unclear, due to restrictions on proprietary information, how the acid pretreated LCH #1 and #2 differ. These results also suggest that the driving difference between strain profiles is the pretreatment method, with lesser impact of the type of biomass used. Upon further inspection of individual strain performance, we found the BY4741 lab strain and CEN.PK2 (**Fig. 1A**, green arrows) clustered with a group of strains that generally grew slowly in most hydrolysates; this group also included many sake-producing strains, several bread-making strains, and strains isolated from natural fermentations. One cluster contains 11 strains with high growth-rate ranks across most hydrolysate conditions. These include isolates from soil (DBVPG1373), fermentation (Y9, Y12), lab (FL100), banana (YB-210), oak (YPS1009), clinical (YJM440, YJM653, YJM978, YJM981) and unknown (NCYC361) sources. Given our interest in understanding the genetic determinants of xylose fermentation from AFEX-pretreated LCHs, we focused on YB-210 ([33], also referred to as GLBRCY0 [40]), that displayed broad tolerance across the hydrolysates tested but ranked highest in growth for alkaline pretreatments. YB-210 grew robustly in AFEX, AHP and dilute acid pretreated LCHs, and less robustly in IL pretreated LCH (**Figs. 1B and C**) under aerobic conditions. Furthermore, YB-210 displays higher tolerance to elevated temperature [34] and inhibitors found in AHP-pretreated LCHs [25]. Therefore, the YB-210 strain background was selected for metabolic engineering and evolution of anaerobic xylose fermentation. As an added benefit of this work, other researchers can utilize this phenotypic dataset to select from publically available strains tailored for tolerance to their pretreated biomass of interest (*e.g.*, YPS163 or YPS1000 for dilute acid pretreated wheat straw).

### Two-stage directed evolution using YB-210 harboring xylose isomerase, transaldolase and xylulokinase permitted anaerobic xylose fermentation

After phenotypic observations revealed its stress tolerant properties, the YB-210 was engineered for xylose metabolism by insertion of an expression cassette containing the *XYL1* (xylose reductase, XR), *XYL2* (xylitol dehydrogenase, XDH) and *XYL3* (xylulokinase) genes from *Sch. stipitis* [40], and then aerobically adapted on xylose. One evolved isolate (GLBRCY73, or Y73) that displayed improved xylose consumption rates in both lab media (**Fig. S12A**) and AHP SG hydrolysate (ASGH) was selected for further study [25]. We first examined the ability of Y73 to ferment xylose under controlled anaerobic conditions in N_2_-sparged bioreactors containing YPDX in lab medium (**Fig. S12B**) or ACSH (**Fig. S12C**). Although the Y73 strain aerobically consumed ∼40% of the xylose in 48 h, it anaerobically fermented <20% and <5% of the xylose in YPDX and ACSH, respectively, in the same time period. These results indicated that the XR-XDH engineered strain is severely impaired for anaerobic fermentation of xylose, particularly in ACSH, relative to aerobic culturing and suggested that this engineered strain would not be useful for our goals of better understanding anaerobic xylose fermentation. Attempts to adapt Y73 for anaerobic growth on xylose yielded no improved clones.

Similar to our observations with the GLBRCY73 strain, reduced anaerobic xylose consumption rates from other *S. cerevisiae* strains expressing *Sch. stipitis* XR-XDH enzymes have been reported. This limitation is likely due to redox cofactor imbalance. Heterologous engineering of *Sch. stipitis XYL1*, which primarily utilizes NADPH as its reducing cofactor, and *XYL2*, which uses NAD^+^ as its oxidizing cofactor, introduces non-regenerative cycles in *S. cerevisiae* that are rapidly imbalanced in the absence of oxygen [4]. A possible alternative to circumvent this problem is to utilize xylose isomerase (XI) [54]–[57], which catalyzes the conversion of xylose into xylulose without cofactors, in place of *XYL1* and *XYL2*. We therefore re-engineered the diploid YB-210 strain with an expression cassette containing the *ScTDH3* promoter upstream of the *Clostridium phytofermentans* xylose isomerase (*CpxylA*), which has been shown to confer anaerobic xylose fermentation onto *S. cerevisiae* after additional genetic modifications [54]. Our cassette also included *ScTAL1*, a pentose phosphate pathway transaldolase enzyme that can improve xylose metabolism when overexpressed [58], [59], and *SsXYL3* driven by the *ScPGK1* and *ScTEF2* promoters, respectively. Finally, to simplify future genomic resequencing of evolved descendants and to rapidly uncover beneficial recessive traits during directed evolution, we isolated one haploid spore, named GLBRCY22-3 (Y22-3), which maintained the *TAL1-xylA-XYL3* gene expression cassette.

**Figure 2:**
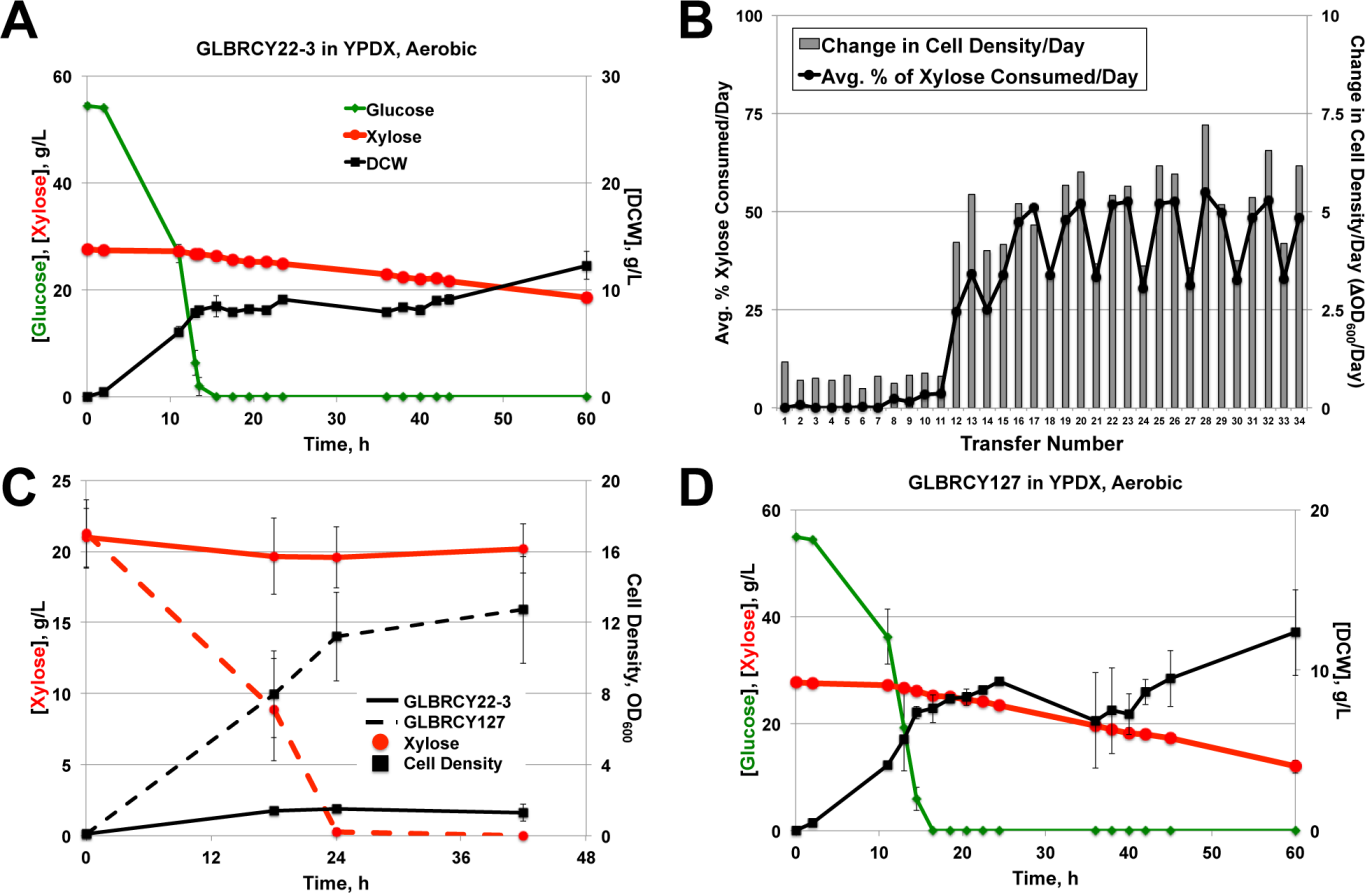
The GLBRCY127 strain developed by directed engineering with xylose isomerase coupled with batch evolution can rapidly consume xylose aerobically. Average sugar consumption and cell growth of unevolved GLBRCY22-3 strain engineered with *ScTAL1*, *CpxylA* and *SsXYL3* cultured in bioreactors containing YPDX media and sparged with air from biological duplicates is shown (**A**). Indicated components were quantified from media samples at times from initial inoculation. In (**B**), the average percentage of xylose consumed and change in cell density per day are plotted for each transfer during the adaption of the Y22-3 strain in YP media containing 0.1% glucose and 2% xylose. The pattern of lower % of xylose consumed and change in cell density per day during every third transfer is due to reaching saturated growth prior to transfer. Average extracellular xylose concentrations and cell density measurements from parental Y22-3 and evolved Y127 strains grown aerobically in culture tubes with YPX media from three independent biological replicates are plotted in (**C**). In (**D**), evolved isolate Y127 was cultured in the same conditions as in (**A**), and samples measurements taken in an identical manner.

To assess whether the engineered Y22-3 strain could metabolize xylose, Y22-3 was cultured aerobically in bioreactors with YPDX medium. Concentrations of extracellular glucose, xylose and dry cell weights were measured over the course of the fermentation (**Fig. 2A**). The Y22-3 strain consumed less than half of the xylose within 64 h, which was significantly less than the Y73 strain. Thus, the Y22-3 strain was subjected to aerobic batch selection in YP medium containing 0.1% glucose and 2% xylose and without exogenous mutagens. Anaerobic batch selection of Y22-3 in YP medium containing 0.1% glucose and 2% xylose was also performed without observing appreciable cell growth and was therefore abandoned, while adaptation in ACSH was not performed because high glucose concentrations (60 g/L in 6% glucan loading ACSH) and diauxic xylose consumption would prevent selection for improved growth on xylose present at 30 g/L. For the first seven transfer cycles in YP-0.1% glucose and 2% xylose, each of which took place over 3–4 day periods, the culture grew at rates of ∼1 generation per day with limited xylose consumption from the medium (**Fig. 2B**), suggesting that most of the growth was on glucose. Over the 8^th^ to 11^th^ transfers, slightly greater xylose consumption was observed, but without substantially faster cell growth rates. By the 12^th^ transfer and beyond, the culture adapted to xylose, consuming all of the sugars within the 2–4 day passaging cycle and reaching saturated growth. After the 34^th^ transfer (∼115 generations), the culture was plated and single clones were screened for growth on xylose-containing medium. One clone, GLBRCY127 (Y127), displayed rapid aerobic growth in YPX by 96-well plate assay (data not shown), and was evaluated for aerobic xylose consumption in culture tubes containing YPX medium (**Fig. 2C**) or bioreactors containing YPDX medium (**Fig. 2D**). Compared to the Y22-3 strain, the evolved Y127 isolate displayed 15 to 17-fold faster absolute and specific xylose consumption rates than the Y22-3 parent in aerobic YPDX medium (**Figs. 2A, D** and **Table 2**). These results indicate that the Y127 isolate evolved from Y22-3 with properties allowing faster aerobic xylose consumption.

**Table 2:** Fermentation kinetic profiles for engineered and evolved *S. cerevisiae* strains.

Media	Aerobic YPDX			Anaerobic YPDX			Anaerobic ACSH		
Strain	Y73	Y22-3	Y127	Y73	Y127	Y128	Y73	Y127	Y128
Absolute xyloseconsumption rate^1^	0.47±0.02	0.17±0.003	0.31±0.01	0.30±0.02	0.094±0.031	1.68±0.06	0.28±0.01	0.04±0.03	0.52±0.01
Specific xyloseconsumption rate^2^	0.039±0.001	0.019±0.000	0.036±0.01	0.066±0.010	0.016±0.007	0.27±0.06	0.059±0.01	0.013±0.01	0.18±0.02
% of theoretical ethanolyield for consumedsugars^3^	ND	ND	ND	80.1±1.4	86.0±2.5	87.5±1.1	72.1±10.4	78.9±14.3	77.2±6.4
% of theoretical ethanolyield for consumedxylose^4^	ND	ND	ND	9.8±5.2	ND*	86.2±15.6	20.9±18.9	ND*	71.5±7.0
Y_x/glc_ 5	ND	ND	ND	0.08±0.00	0.09±0.00	0.11±0.02	0.066±0.012	0.045±0.004	0.05±0.01
Y_glycerol/glc_ 6	ND	ND	ND	0.10±0.00	0.07±0.00	0.08±0.01	0.051±0.002	0.038±0.006	0.04±0.00

ND, Not Determined for aerobic conditions; ND*, Not Determined – no ethanol produced.

1 In g xylose consumed/L/h.

2 In g xylose consumed/g DCW/h.

3 Calculated from the maximum ethanol concentration produced divided by the consumed xylose concentration at that time.

4 Calculated from the ethanol concentration produced between two time points after glucose depletion.

5 Yield of g DCW/g glucose consumed calculated at or near the time of glucose depletion and prior to xylose consumption. No cell growth was observed during xylose consumption.

6 Yield of g glycerol/g glucose consumed calculated at or near the time of glucose depletion and prior to xylose consumption.

**Figure 3:**
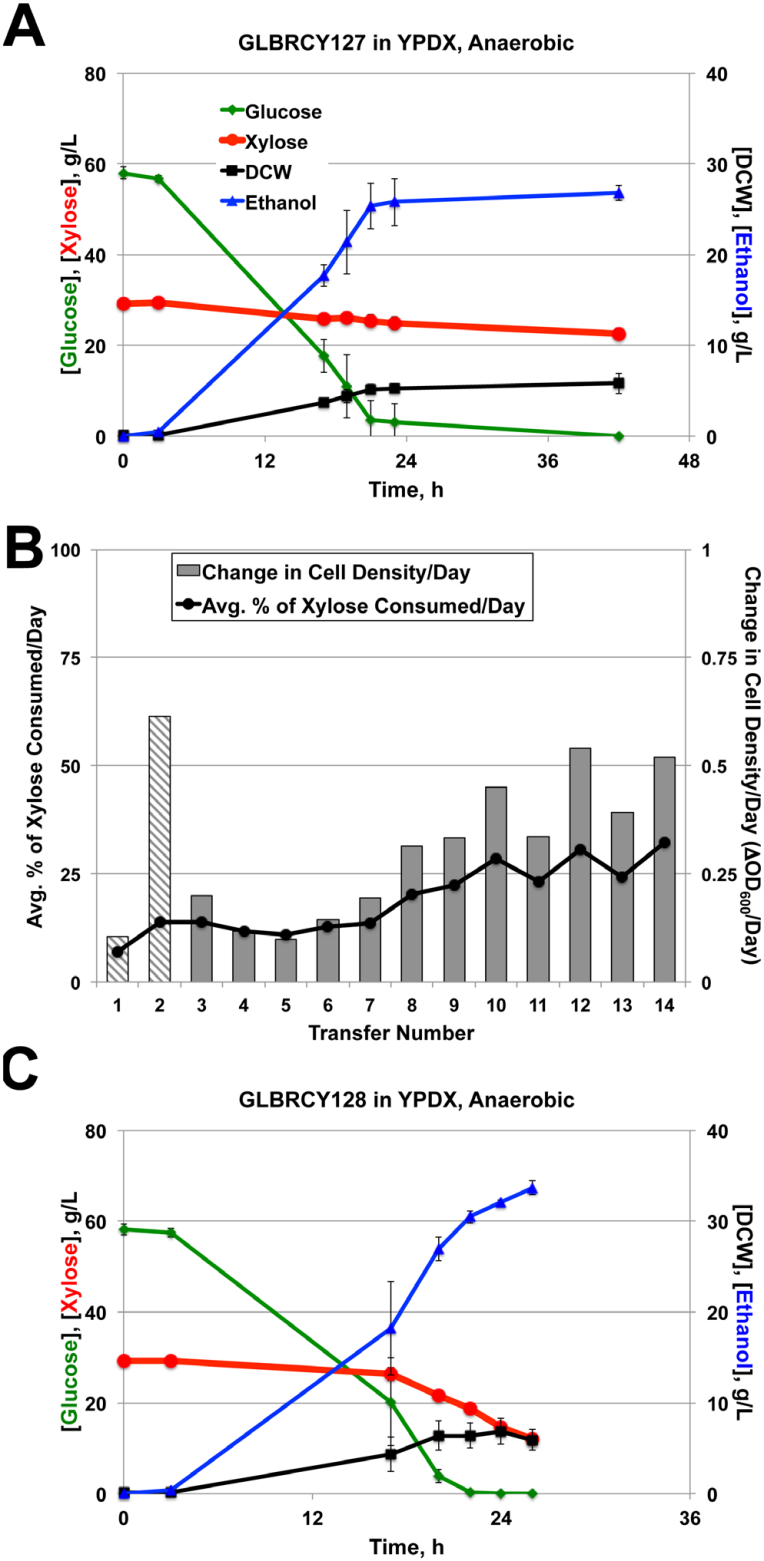
Second stage anaerobic adaptation on xylose enabled rapid xylose fermentation by evolved GLBRCY128 isolate. Average fermentation kinetic profiles of the GLBRCY127 strain cultured in bioreactors containing YPDX media and sparged with nitrogen from biological duplicates are shown (**A**). Average concentrations with standard deviations of indicated compounds were quantified from media samples at times from initial inoculation. In (**B**), the percentage of xylose consumed and change in cell density per day is plotted for each transfer during the anaerobic adaptation of Y127 in YP media containing 0.1% glucose and 2% xylose. In the first two transfers (hatched bars), Tween-80 and ergosterol were added to the media. In (**C**), evolved isolate Y128 was cultured in biological duplicate under the same conditions as in (**A**), and samples measurements taken in an identical manner.

We next assessed the ability of the Y127 strain to ferment xylose anaerobically in bioreactors sparged with N_2_. Similar to the XR-XDH engineered Y73 strain, the aerobically evolved Y127 strain displayed limited xylose fermentation from YPDX medium, consuming less than 30% of the total xylose within 42 h, and did not appear to convert the small amount of consumed xylose into ethanol (**Fig. 3A**). This suggested that, like Y73, the Y127 strain was not capable of effectively fermenting xylose in the absence of oxygen. In an attempt to overcome this barrier, we performed a second round of batch selection of the Y127 strain cultured in YP medium containing 0.1% glucose and 2% xylose under completely anaerobic conditions (**Fig. 3B**). During the first two transfers, 40 µg/L ergosterol and 4 g/L Tween-80 were added to support anaerobic growth, but then omitted for all successive transfers. For the first six transfers, the cell population doubled approximately twice per week. After the 6^th^ transfer, the culture began to grow faster and consumed a greater percentage of the total xylose per day. After reaching 33 generations at the 10^th^ transfer, the culture appeared to plateau in anaerobic growth and xylose consumption rate. After the 14^th^ transfer (∼47 generations), the culture was plated and colonies were screened for fastest growth rate in YPX medium by 96-well plate assay. One clone (GLBRCY128, Y128) displaying rapid anaerobic growth on xylose (data not shown) was then evaluated in bioreactors containing YPDX medium sparged with N_2_. In contrast to Y127 (**Fig. 3A**), the Y128 strain rapidly fermented xylose in the absence of oxygen, during which time the extracellular ethanol concentration increased (**Fig. 3C**). Consistent with this result, Y128 exhibited higher absolute and specific xylose consumption rates in anaerobic YPDX medium than the Y127 strain (**Table 2**). These results indicate that the two-stage directed evolution yielded Y127 and Y128 strains with enhanced aerobic and anaerobic xylose metabolism, respectively.

### The evolved Y128 strain can anaerobically convert xylose into ethanol faster than the parental strains

**Figure 4:**
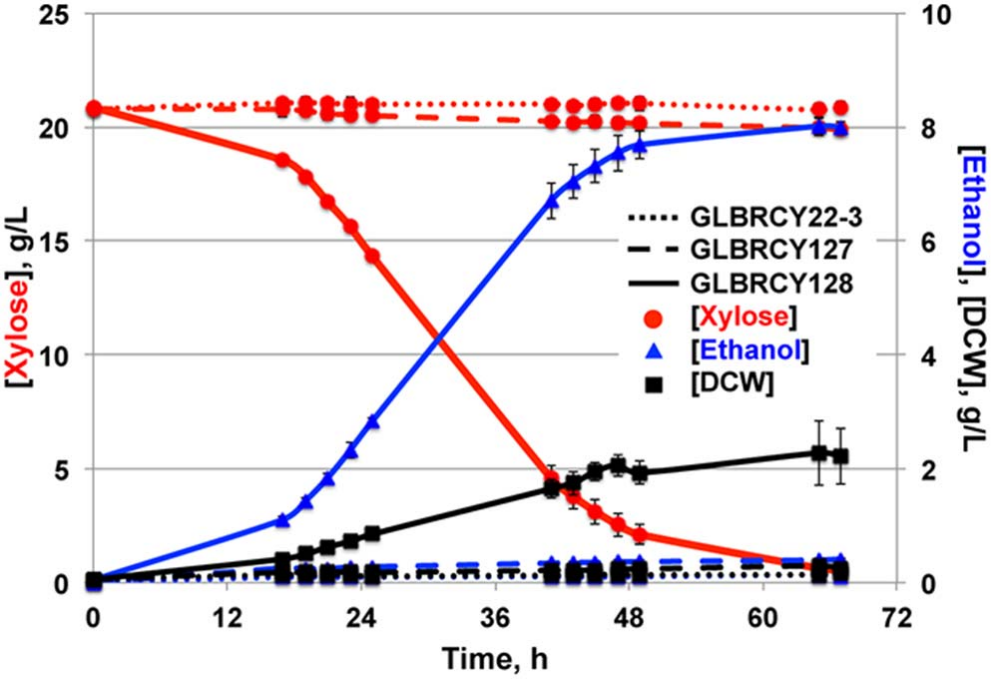
GLBRCY128 can anaerobically ferment xylose from YPX medium into ethanol faster than its parental strains. Fermentation kinetic profiles comparing Y22-3, Y127 and Y128 strains cultured anaerobically in bioreactors containing YPX medium and sparged with nitrogen are shown. Average concentrations with standard deviations of xylose, ethanol and DCW concentrations in the media were calculated from independent biological triplicates.

While the YPDX media matched the predominant sugar concentrations found in 6% glucan loading ACSH, the inclusion of 60 g/L glucose clouded our ability to directly compare the abilities of our engineered and evolved strains in the anaerobic conversion of xylose into ethanol. Therefore, we performed anaerobic fermentations with Y22-3, Y127 and Y128 strains in bioreactors containing YP media with 20 g/L xylose, and quantified xylose consumption and ethanol production from the media over time. For Y128, there was production of ethanol and cell biomass with simultaneous depletion of xylose from the media (**Fig. 4**). In contrast, both Y22-3 and Y127 strains produced less than 1 g/L ethanol (**Table 3**) and consumed less than 1 g/L xylose (**Fig. 4**) by the end of fermentation. Importantly, these differences in xylose consumption and ethanol production showed that Y128 consumed xylose significantly faster and produced ethanol at a higher yield and titer than Y22-3 and Y127 (**Table 3**). These results further support that the evolved Y128 strain displays marked improvement in anaerobic xylose fermentation compared to its parental strains.

**Table 3:** Comparison of anaerobic fermentation kinetics for Y22-3, Y127 and Y128 in YPX medium.

Strain	Y22-3	Y127	Y128
Absolute xylose consumption rate^1^	ND	0.101±0.004	0.596±0.028
Specific xylose consumption rate^2^	ND	0.515±0.009	0.587±0.038
% of theoretical ethanol yield for consumed xylose^3^	ND*	ND*	77.7±0.6
% of theoretical ethanol yield from total xylose^4^	1.2±0.8	3.9±0.2	74.7±0.5
Avg. final ethanol titer^5^	0.11±0.03	0.42±0.02	8.0±0.1

ND, Not Determined – low xylose consumption; ND*, Not Determined – no ethanol produced and low xylose consumption.

1 In g xylose consumed/L/h.

2 In g xylose consumed/g DCW/h.

3 Calculated from the maximum ethanol concentration produced divided by the consumed xylose concentration at that time.

4 Calculated from the maximum ethanol concentration produced divided by the starting xylose concentration.

5 In g ethanol/L.

### The evolved Y128 strain can anaerobically convert xylose from ACSH into ethanol

**Figure 5:**
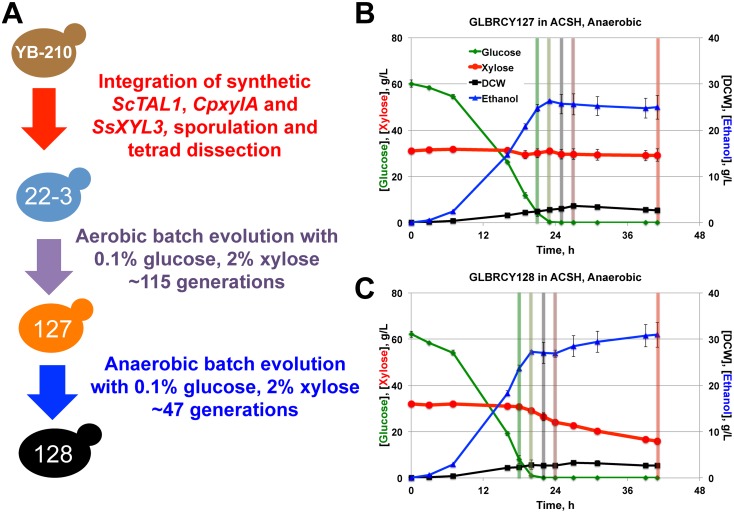
GLBRCY128 can anaerobically ferment xylose from ACSH. A diagram summarizing the engineering and evolution of the YB-210 strain into the evolved Y128 strain is provided in (**A**). Fermentation kinetic profiles of the Y127 (**B**) and Y128 (**C**) strains cultured in bioreactors containing ACSH and sparged with nitrogen from biological duplicates are shown. Average concentrations and standard deviations of indicated components were quantified from media samples at times from initial inoculation. Vertical colored bars indicate time points at which samples were taken for metabolomic analysis described in Fig. 7A–D.

Within a relatively small number of generations (∼162 in total) without exogenous mutagens, our two-stage evolution yielded a *xylA*-engineered *S. cerevisiae* strain with the ability to consume xylose anaerobically in lab medium (summarized in **Fig. 5A**). Although the Y128 genetic background originated from a hydrolysate-tolerant strain, Y128 could have lost stress-tolerance traits during the course of xylose evolution. If so, Y128 might not have been able to convert xylose in lignocellulosic hydrolysates into ethanol anaerobically, which was a chief goal of this research. Previous studies have shown that yeast strains able to ferment xylose rapidly in lab medium are severely impaired in LCHs [34]. Therefore, we assessed the abilities of the Y127 and Y128 strains to ferment sugars anaerobically from ACSH in bioreactors sparged with N_2_. Y22-3 was not used in this study, as it clearly does not significantly metabolize xylose aerobically (**Fig. 2**) or anaerobically (**Fig. 4**). Similar to our observations with YPDX lab medium, both Y127 and Y128 strains fermented glucose rapidly (**Figs. 5B and C**). However, the Y128 strain, but not Y127, consumed most of the xylose (∼50% within 44 hrs) once glucose was depleted from ACSH. Indeed the absolute and specific xylose consumption rates for Y128 were approximately 10-fold higher than Y127 (**Table 2**). This also resulted in a higher yield in ethanol from xylose for Y128 compared to Y127 and Y73 (**Table 2**). Importantly, the ethanol yield from glucose for Y128 was similar to Y127, suggesting that the anaerobic xylose evolution had little impact on the ability of Y128 to convert glucose into ethanol (**Table 2**). Because Y128 ferments more xylose than Y127 anaerobically, this resulted in a higher ethanol titer for Y128 (**Fig. 5B and C**). Thus, despite the fact that evolution for xylose conversion occurred in lab medium lacking the inhibitors found in LCHs, the Y128 strain could effectively ferment xylose from an industrially relevant pretreated LCH in the absence of oxygen. This ability may be due in part to the innate hydrolysate-tolerant properties of the YB-210 genetic background.

### Rapid xylose consumption by GLBRCY127 and Y128 is dependent upon *xylA* and *TAL1* expression

**Figure 6:**
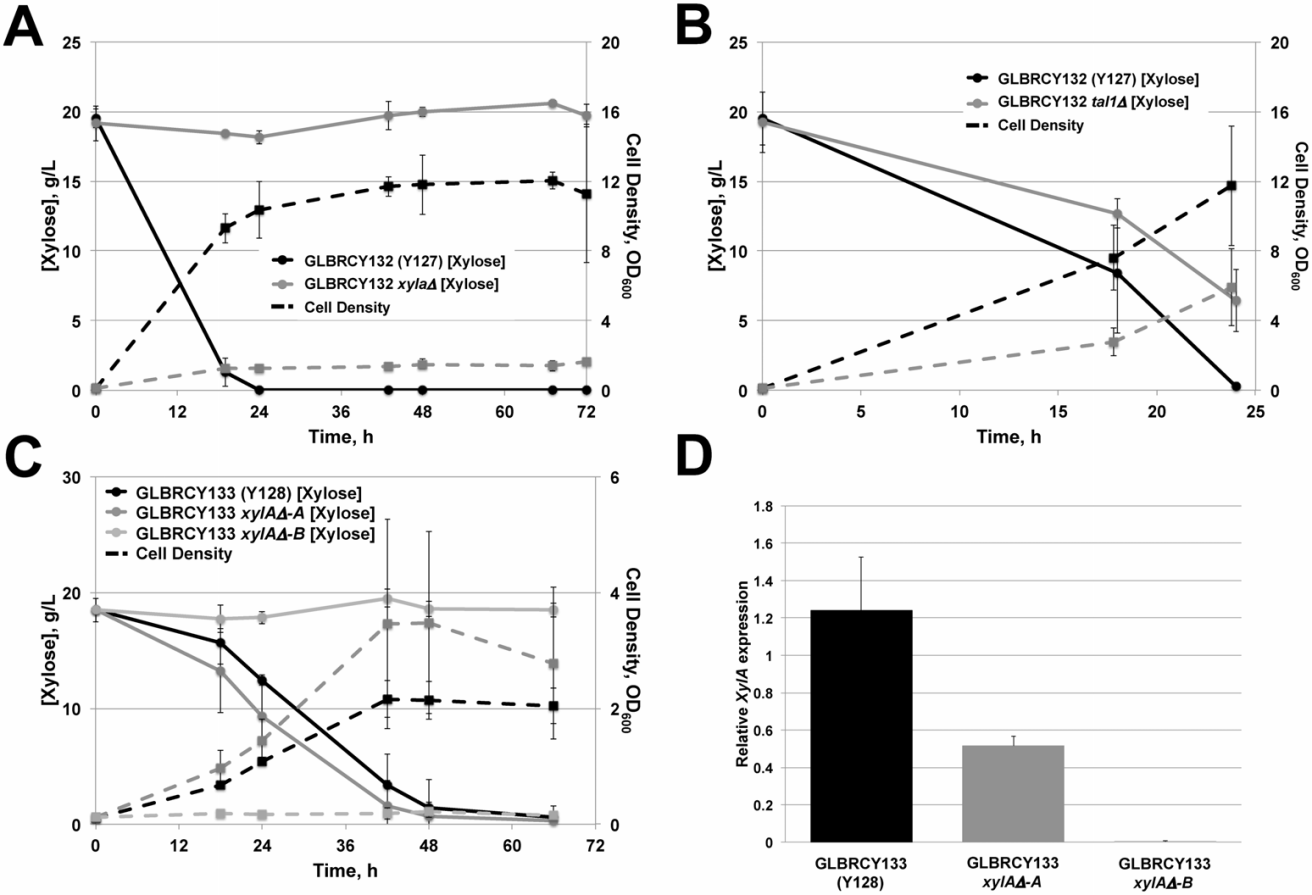
The xylose consumption phenotypes of the evolved Y127 and Y128 strains are dependent upon *CpxylA* and *ScTAL1.* Extracellular xylose concentrations (solid lines) and cell density (dashed lines) were measured by YSI instrument and OD_600_ readings, respectively, from cultures containing KanMX marker rescued versions of (**A**) GLBRCY127 (Y132) and GLBRCY132 *xylAΔ* or (**B**) Y132 and Y132 *tal1Δ* strains inoculated in aerobic YPX media. In (**C**), extracellular xylose concentrations (solid lines) and cell density (dashed lines) were measured as in (**A**, **B**) for KanMX marker rescued GLBRCY128 (Y133) and two independent GLBRCY133 *xylAΔ* strains inoculated in anaerobic YPX media. These selection marker-rescued Y128 strains were cultured in YPD media and total RNA isolated from a single time point. Expression of *CpxylA* was then quantified and normalized to *ScERV25* RNA levels by qPCR. The bar graph in (**D**) displays the average values and standard deviations for *CpxylA* RNA from three independent biological replicates.

After clearly establishing the faster xylose consumption phenotypes of the Y127 and Y128 strains relative to the parental Y22-3 strain, we next wanted to better understand the potential genetic mechanisms by which these strains could have evolved. One obvious possibility would be through mutations in the engineered genes *xylA*, *TAL1* and *XKS1* that increase their expression or activities. However, when we sequenced the engineered gene cassette, no DNA sequence differences were identified. An alternative possibility is that the Y127 or Y128 strains obtained gain-of-function mutations in native genes that code for xylose metabolism enzymes, which are normally expressed at low levels or lack sufficient activities for rapid flux into the pentose phosphate pathway [4]. The *S. cerevisiae* genome contains a number of putative enzymes with xylose reductase activities, including Gre3p [60], [61], and xylitol dehydrogenases, including an ineffective *XYL2* homolog [62] and *XDH1*, which is present only in some wild *S. cerevisiae* strains and confers detectable xylose consumption [63]. Thus, one possible model for the evolution of Y127 and Y128 is that genetic changes in one or more of these genes allowed for improved xylose consumption independent of engineered *xylA* and *TAL1*. We examined this possibility by first excising the loxP-KanMX-loxP selection marker from the engineered cassette from Y22-3, Y127 and Y128 to generate antibiotic-sensitive GLBRCY36, GLBRCY132 and GLBRCY133, respectively. We then deleted *xylA* from the engineered cassette of the Y127 marker rescued strain (Y132) and assessed its ability to consume xylose aerobically. Indeed, the Y132 *xylA* ***Δ*** strain was ablated of its ability to consume or produce cell biomass from xylose (**Fig. 6A**). In contrast, deletion of *TAL1* from the engineered expression cassette, but not endogenous *TAL1*, in Y132 reduced the rate of xylose metabolism but did not impact the final amount of xylose consumed or the cell biomass produced from xylose (**Fig. 6B**), suggesting that the additional copy of engineered *TAL1* was important for determining the rate of xylose consumption but was not essential. In addition, we confirmed two independent marker rescued Y128 (Y133) *xylAΔ* transformants that displayed separate xylose consumption phenotypes. Consistent with the Y132 *xylAΔ* strain, the Y133 *xylAΔ-B* strain could not consume xylose aerobically (data not shown) or anaerobically (**Fig. 6C**). Interestingly, the Y133 *xylAΔ-A* strain fermented xylose at a comparable rate to the Y133 strain. This suggested the possibility that the *xylA* gene was duplicated in *cis*, which could explain why the replacement of *xylA* with the KanMX deletion cassette could be verified by PCR. Indeed, when we compared *xylA* RNA expression in the two Y133 *xylAΔ* strains to the Y133 strain by qPCR, we found that Y133 *xylAΔ-A* expressed half as much *xylA* as Y133, whereas no *xylA* RNA was detected in the Y133 *xylAΔ-B* strain (**Fig. 6D**). Although this result does not rule out possible genetic changes in endogenous xylose metabolizing enzymes, our findings together suggest that the evolved xylose consumption phenotypes in Y127 and Y128 are dependent, at least in part, upon the engineered *xylA* and *TAL1* genes.

### Y128 accumulates higher intracellular concentrations of xylose metabolic intermediates but little xylitol

**Figure 7:**
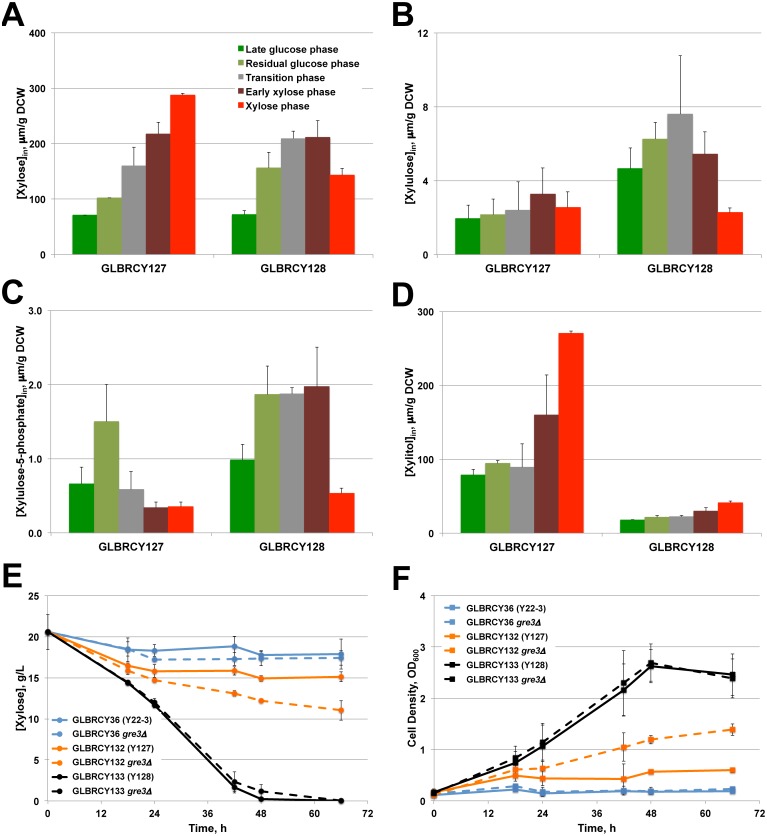
Y128 has a mutation in *GRE3* that reduces xylitol production and contributes towards anaerobic xylose fermentation. Fermentation samples were taken at the indicated time points marked by vertical colored bars in Fig. 5B and C. Cells were then filter-captured, briefly washed and then intracellular metabolites extracted. Average concentrations of xylose (**A**), xylulose (**B**), xylulose-5-phosphate (**C**) and xylitol (**D**) from independent duplicate fermentations were determined by reverse phase ion pairing HPLC-ESI coupled with MS/MS or gas chromatography (see Materials and Methods). Average concentrations and standard deviations are based on two biological replicates. Y22-3, Y127 and Y128 strains with KanMX selection markers excised (Y36, Y132 and Y133, respectively) and with or without deletion of *GRE3* were cultured under anaerobic conditions in YPX media. Samples were taken at the indicated time points to measure xylose concentrations (**E**) or cell density (**F**). Average values and standard deviations were calculated from biological triplicates.

The data presented thus far suggest Y128 has an evolved ability to ferment xylose anaerobically in lab medium (**Figs. 3C and D**) and ACSH (**Fig. 5C**). To investigate how the evolved strains anaerobically ferment xylose, we analyzed the intracellular concentrations of xylose catabolism intermediates: xylose, xylitol, xylulose and xylulose-5-phosphate (X5P). During the anaerobic fermentation of ACSH by the Y127 and Y128 strains (**Figs. 5B and C**), cells were captured from the bioreactors using rapid vacuum filtration at five different time points spanning the glucose and xylose consumption phases; two samples were taken when glucose was detected in the hydrolysate (indicated by vertical lines in shades of green); one sample was taken during the transition to xylose after glucose was undetectable (grey vertical line), and two final samples were taken when xylose consumption (vertical lines in shades of red) was evident. Cell samples for Y127 and Y128 fermentations were taken at comparable sugar concentrations, whereas the last two samples were taken at the equivalent amount of time after the transition time point. During the course of the ACSH fermentation, the Y127 cells accumulated xylose (**Fig. 7A**), whereas xylulose, which is the product of isomerization of xylose by *CpxylA*, did not change (**Fig. 7B**). In contrast, Y128 peaked in intracellular xylose and xylulose levels during the transition phase, after which intracellular xylose decreased slightly at the end of the fermentation coincident with extracellular xylose depletion (**Figs. 7A and B**). The final metabolite of the engineered xylose metabolism pathway, X5P, peaked in intracellular concentration between the residual glucose and early xylose metabolism phases in Y128, whereas for Y127, X5P briefly peaked in the residual glucose phase, then decreased to low levels for the remainder of the fermentations (**Fig. 7C**). These patterns of intracellular xylose, xylulose and X5P accumulation, along with the simultaneous depletion of extracellular xylose and accumulation of ethanol (**Fig. 5C**), suggested the possibility that the xylose consumed by Y128 from ACSH was metabolized through the engineered *xylA* pathway at higher flux than in Y127.

The higher concentrations of catabolized pentose intermediates along with the faster xylose consumption rates (**Tables 2** and **3**) suggest two possible biochemical mechanisms in the evolution of Y128. One possibility is that the apparent increase in xylose consumption from the hydrolysate is due solely to improved xylose transport, without further metabolic conversion of xylose to other products. However, the higher ethanol titer achieved by Y128 coincident with differences in intracellular concentrations of metabolized xylose intermediates supports alternate models in which the evolved Y128 strain has more active xylose catabolism or pentose phosphate enzymes, or both, relative to its Y127 parent, possibly allowing for greater flux of xylose to ethanol.

The accumulation of internalized xylose and unchanging levels of xylulose in Y127 during the ACSH fermentation, along with the paltry change in extracellular xylose levels, suggest a metabolic bottleneck in xylose isomerase activity. Xylitol is a known inhibitor of xylose isomerase [64], and it is know that deletion of *GRE3*, which encodes a reductase enzyme that can convert xylose into xylitol, in *S. cerevisiae* engineered with xylose isomerase improved xylose fermentation [17], [55], [57], [61], [65]. Therefore, we quantified and compared the intracellular levels of xylitol in Y127 and Y128 during ACSH fermentation. Strikingly, we observed a severe reduction in intracellular xylitol levels for Y128 at all time points compared to Y127, which accumulated xylitol over time (**Fig. 7D**). This suggested that one difference between the Y127 and Y128 could be an evolved mutation that alters *GRE3* activity or expression. Thus, we sequenced the *GRE3* open reading frames of the Y22-3, Y127 and Y128 strains. We found a single nucleotide polymorphism (SNP) in the Y128 strain producing a G-to-A mutation relative to both Y22-3 and Y127, which changed the alanine at amino acid residue 46 to threonine (A46T) in Gre3p. This residue is conserved in other *S. cerevisiae* strains, as well as in many other yeast species including *Saccharomyces arboricola*, *S. kudriavzevii*, *Candida* and *Kluyveromyces*, and resides within the aldo-ketoreductase catalytic domain, suggesting that the A46T substitution likely impairs Gre3p xylose reductase activity. Although others have rationally deleted *GRE3* from strains to improve XI-mediated xylose metabolism prior to evolution, our observation of a spontaneous *GRE3* mutation acquired from directed evolution of a *xylA*-engineered yeast strain confirms its importance in the xylose metabolism bottleneck.

Taken together, our results suggest that the *gre3^A46T^* mutation in Y128 may cause a partial or complete loss of Gre3p function, which in turn reduces xylitol production and thus minimizes inhibition of *CpxylA*. To further confirm the possibility that loss of Gre3p activity improves anaerobic xylose fermentation, we deleted *GRE3* in the marker rescued Y22-3 (renamed Y36), Y127 (renamed Y132) and Y128 (renamed Y133) strains and compared their *in vitro* xylose reductase activities and abilities to ferment xylose in the absence of oxygen. First, xylose reductase activities from extracts generated from selection-marker rescued Y127 (Y132), Y132 *gre3* ***Δ***, marker rescued Y128 (Y133) and Y133 *gre3* ***Δ*** cells were determined (**Fig. S13**). Extracts from the Y133 strain, which harbors the *gre3^A46T^* mutation, and *gre3* ***Δ*** strains displayed similar decreased xylose reductase activity compared toY132, which contains wild-type *GRE3*. This result further supported our *in vivo* observations that strains containing the *gre3^A46T^* mutation behave biochemically similar to strains lacking *GRE3*. Finally, we found that the Y132 *gre3Δ* strain could anaerobically consume xylose (**Fig. 7E**) and grow faster than the parental Y132 strain (**Fig. 7F**), but not nearly as fast as Y133. This suggests that the Y128 strain contains mutations in addition to *gre3^A46^* ^T^ that aid anaerobic xylose fermentation. Most importantly, there were no differences in the xylose consumption or growth rates between the Y133 and Y133 *gre3Δ* strains, which is consistent with the *gre3^A46 T^* mutation resulting in a loss of function. Finally, deletion of *GRE3* in the Y36 parental strain had no effect, further indicating that loss of *GRE3* function alone cannot confer anaerobic xylose fermentation. Together, these *in vitro* and *in vivo* results suggest that the Y128 strain evolved a loss-of-function mutation in *GRE3*, which along with other unknown mutations, contributed to improved xylose utilization by reducing the production of inhibitory xylitol.

## Conclusions

Here, we report the development of an engineered haploid *S. cerevisiae* strain with the evolved ability to ferment xylose anaerobically in lab media and LCH. Although yeast strains with improved anaerobic xylose fermentation in lab media and pretreated LCHs have been reported, most are derived from polyploid industrial strains with robust tolerance traits [12], [17], [25], [30]–[32]. Although polyploidy can compensate for detrimental recessive alleles, the multiple gene copies present in these industrial strains make mapping and identifying the causal mutations that accelerate xylose conversion difficult. In contrast, we generated the haploid Y128 strain with the ability to rapidly ferment xylose anaerobically, even in the presence of ACSH inhibitors. Thus, haploid Y22-3 and its evolved Y127 and Y128 descendants are ideally suited for comparative analyses to identify gene sequences that contribute to xylose conversion in the presence and absence of oxygen, and to determine metabolomic and transcriptomic differences that underlie their respective phenotypes. As a proof of concept, we used metabolomic data and targeted gene sequencing to identify a loss-of-function mutation in *GRE3*, which we validated by strain reconstruction (**Fig. 7**). Thus, these strains are exciting tools that will provide future opportunities for multi-omic dissection of the molecular bottlenecks to anaerobic xylose fermentation by *S. cerevisiae* under LCH inhibitor stress.

## Supporting Information

Figure S1 **Domesticated strains of** ***S. cerevisiae*** **grow poorly in lignocellulosic hydrolysates.** Representative aerobic growth profiles of lab strains BY4741 (**A**) and CEN.PK2 (**B**) cultured in 96-well plates with hydrolysates made from various pretreated lignocellulose hydrolysates (see Materials and Methods) and supplemented with yeast extract and peptone (YP) are shown by plotting cell density (optical density at 595 nm) every 20 min for 24 h. ACSH; 6% glucan loading AFEX pretreated corn stover hydrolysate, AHP; Alkaline Hydrogen Peroxide pretreatment, IL; Ionic Liquid ([C_2_mim][OAc]) pretreated, Dil. Acid; Dilute Acid pretreated lignocellulosic hydrolysate, SGH; switchgrass hydrolysate, CSH; corn stover hydrolysate.
(TIF)

Figure S2 **Bar graph displaying average growth rates (grey bars) of wild and domesticated** ***S. cerevisiae*** **strains in 6% glucan loading ACSH relative to YPD.** Averages and standard deviations (black bars) are calculated from at least 3 biological replicates. The row location for NRRL YB-210/GLBRCY0 strain used in this study is identified by opposite coloration (average growth rate in black, standard deviation in grey).
(TIF)

Figure S3 **Bar graph displaying average growth rates (grey bars) of wild and domesticated** ***S. cerevisiae*** **strains in 9% glucan loading ACSH relative to YPD.** Averages and standard deviations (black bars) are calculated from at least biological replicates. The row location for NRRL YB-210/GLBRCY0 strain used in this study is identified by opposite coloration (average growth rate in black, standard deviation in grey).
(TIF)

Figure S4 **Bar graph displaying average growth rates (grey bars) of wild and domesticated** ***S. cerevisiae*** **strains in YP-AHP CSH relative to YPD.** Averages and standard deviations (black bars) are calculated from at least biological replicates. The row location for NRRL YB-210/GLBRCY0 strain used in this study is identified by opposite coloration (average growth rate in black, standard deviation in grey).
(TIF)

Figure S5 **Bar graph displaying average growth rates (grey bars) of wild and domesticated** ***S. cerevisiae*** **strains in YP-AHP SGH relative to YPD.** Averages and standard deviations (black bars) are calculated from at least biological replicates. The row location for NRRL YB-210/GLBRCY0 strain used in this study is identified by opposite coloration (average growth rate in black, standard deviation in grey).
(TIF)

Figure S6 **Bar graph displaying average growth rates (grey bars) of wild and domesticated** ***S. cerevisiae*** **strains in YP-6% glucan loading ACSH relative to YPD.** Averages and standard deviations (black bars) are calculated from at least biological replicates. The row location for NRRL YB-210/GLBRCY0 strain used in this study is identified by opposite coloration (average growth rate in black, standard deviation in grey).
(TIF)

Figure S7 **Bar graph displaying average growth rates (grey bars) of wild and domesticated** ***S. cerevisiae*** **strains in YP-detoxified AHP CSH relative to YPD.** Averages and standard deviations (black bars) are calculated from at least biological replicates. The row location for NRRL YB-210/GLBRCY0 strain used in this study is identified by opposite coloration (average growth rate in black, standard deviation in grey).
(TIF)

Figure S8 **Bar graph displaying average growth rates (grey bars) of wild and domesticated** ***S. cerevisiae*** **strains in YP-detoxified AHP SGH relative to YPD.** Averages and standard deviations (black bars) are calculated from at least biological replicates. The row location for NRRL YB-210/GLBRCY0 strain used in this study is identified by opposite coloration (average growth rate in black, standard deviation in grey).
(TIF)

Figure S9 **Bar graph displaying average growth rates (grey bars) of wild and domesticated** ***S. cerevisiae*** **strains in YP-80% dilute acid pretreated hydrolysate #1 relative to YPD.** Averages and standard deviations (black bars) are calculated from at least biological replicates. The row location for NRRL YB-210/GLBRCY0 strain used in this study is identified by opposite coloration (average growth rate in black, standard deviation in grey).
(TIF)

Figure S10 **Bar graph displaying average growth rates (grey bars) of wild and domesticated** ***S. cerevisiae*** **strains in YP-80% dilute acid pretreated hydrolysate #2 relative to YPD.** Averages and standard deviations (black bars) are calculated from at least biological replicates. The row location for NRRL YB-210/GLBRCY0 strain used in this study is identified by opposite coloration (average growth rate in black, standard deviation in grey).
(TIF)

Figure S11 **Bar graph displaying average growth rates (grey bars) of wild and domesticated** ***S. cerevisiae*** **strains in YP-IL SGH relative to YPD.** Averages and standard deviations (black bars) are calculated from at least biological replicates. The row location for NRRL YB-210/GLBRCY0 strain used in this study is identified by opposite coloration (average growth rate in black, standard deviation in grey).
(TIF)

Figure S12 **Hydrolysate-tolerant YB-210/GLBRCY0 engineered with XR/XDH and evolved for aerobic xylose metabolism does not ferment xylose anaerobically.** The YB-210/Y0 strain engineered with *XYL1*, *2* and *3* genes from *S. stipitis* and aerobically-evolved (GLBRCY73) was cultured in bioreactors and evaluated for consumption of xylose in aerobic YPDX (**A**), anaerobic YPDX (**B**) and anaerobic ACSH (**C**) media as described in Materials and Methods. Concentrations (g/L) of glucose (green), xylose (red), dry cell weight (black) and ethanol (blue) are averages and standard deviations from two independent biological replicates.
(TIF)

Figure S13 **GLBRCY133 (Y128) cell extracts display reduced** ***in vitro*** **xylose reductase activity similar to** ***GRE3*** **deletion strains.** The indicated strains were cultured aerobically in YPD, harvested and prepared for *in vitro* xylose reductase activity assays as described in Materials and Methods. Xylose and NADPH were added to each extract, and then rates of change in absorbance at 340 nm were measured to determine the Units of enzymatic activity normalized to mg of total protein in the cellular extract. The graph displays the average percent of *in vitro* xylose reductase activities and standard deviations of indicated strains relative to GLBRCY132 (marker rescued GLBRCY127, which contains wild-type *GRE3*) in biological duplicate.
(TIF)

Table S1 **Wild and domesticated** ***S. cerevisiae*** **strains used in phenotypic growth studies.**
(XLSX)
