# Supplementary figures and images for: Engineering and Two-Stage Evolution of a Lignocellulosic Hydrolysate-Tolerant Saccharomyces cerevisiae Strain for Anaerobic Fermentation of Xylose from AFEX Pretreated Corn Stover
Source: PLoS One. 2014 Sep 15;9(9):e107499. doi: 10.1371/journal.pone.0107499 (PMC4164640; doi:10.1371/journal.pone.0107499)

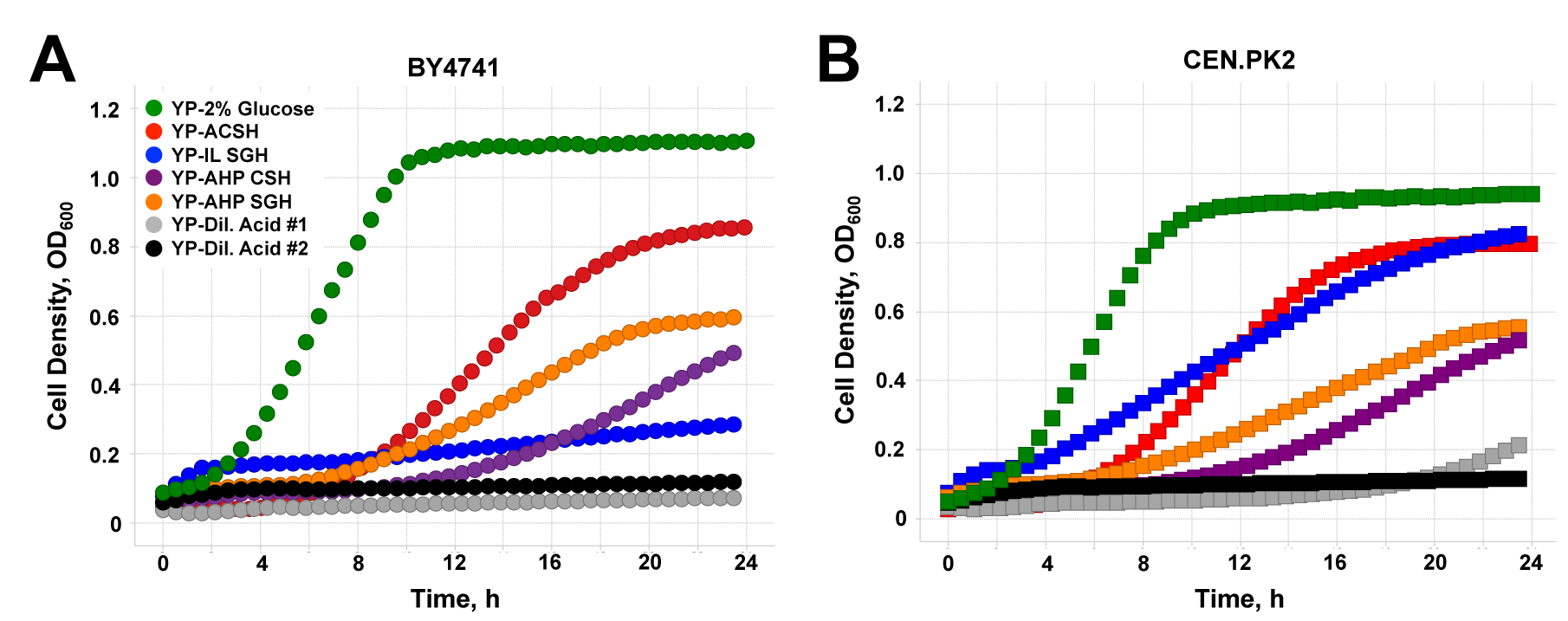

Supplement: Figure S1 — Domesticated strains of S. cerevisiae grow poorly in lignocellulosic hydrolysates. Representative aerobic growth profiles of lab strains BY4741 (A) and CEN.PK2 (B) cultured in 96-well plates with hydrolysates made from various pretreated lignocellulose hydrolysates (see Materials and Methods) and supplemented with yeast extract and peptone (YP) are shown by plotting cell density (optical density at 595 nm) every 20 min for 24 h. ACSH; 6% glucan loading AFEX pretreated corn stover hydrolysate, AHP; Alkaline Hydrogen Peroxide pretreatment, IL; Ionic Liquid ([C2mim][OAc]) pretreated, Dil. Acid; Dilute Acid pretreated lignocellulosic hydrolysate, SGH; switchgrass hydrolysate, CSH; corn stover hydrolysate. (TIF) [file pone.0107499.s001.tif]

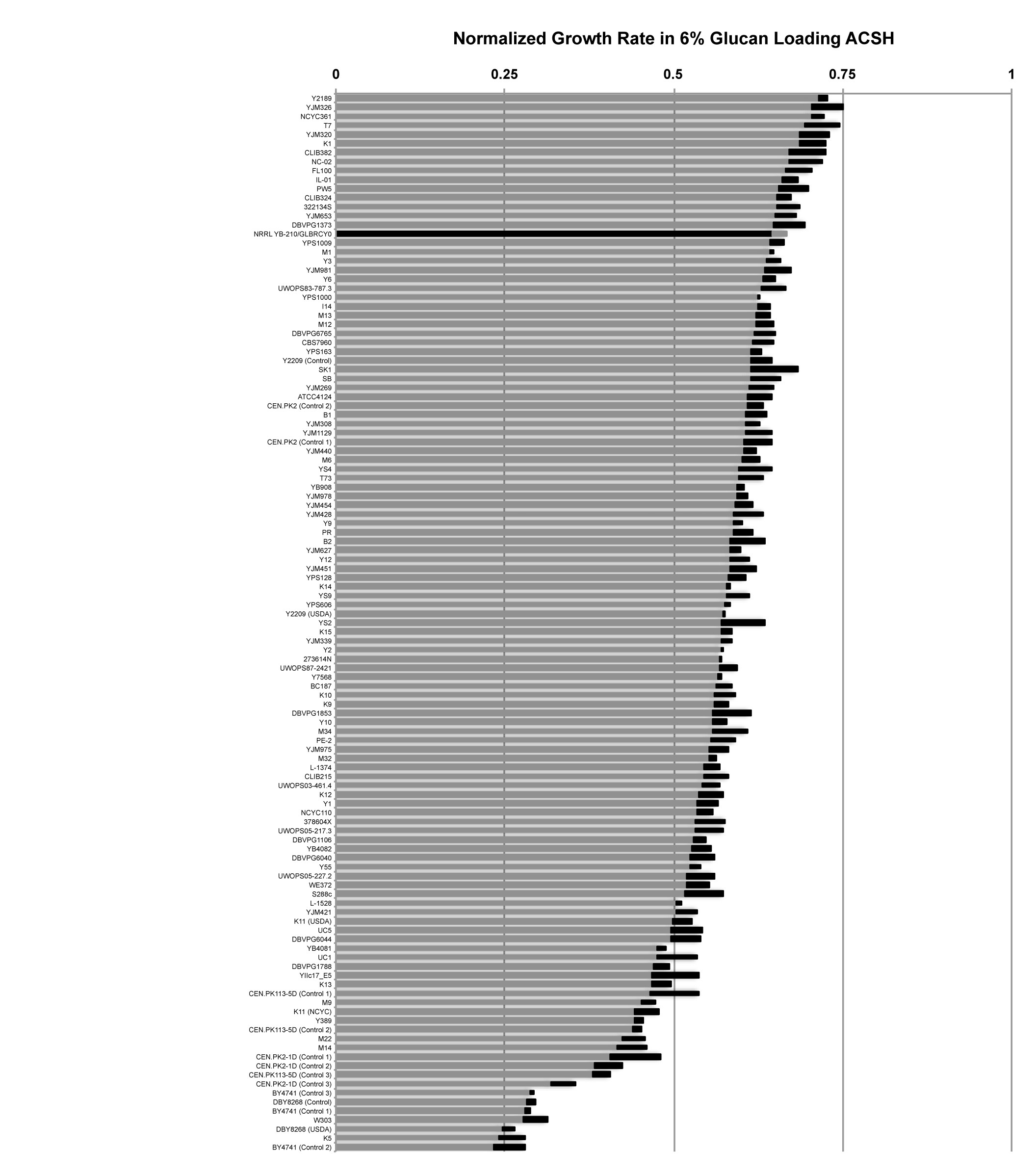

Supplement: Figure S2 — Bar graph displaying average growth rates (grey bars) of wild and domesticated S. cerevisiae strains in 6% glucan loading ACSH relative to YPD. Averages and standard deviations (black bars) are calculated from at least 3 biological replicates. The row location for NRRL YB-210/GLBRCY0 strain used in this study is identified by opposite coloration (average growth rate in black, standard deviation in grey). (TIF) [file pone.0107499.s002.tif]

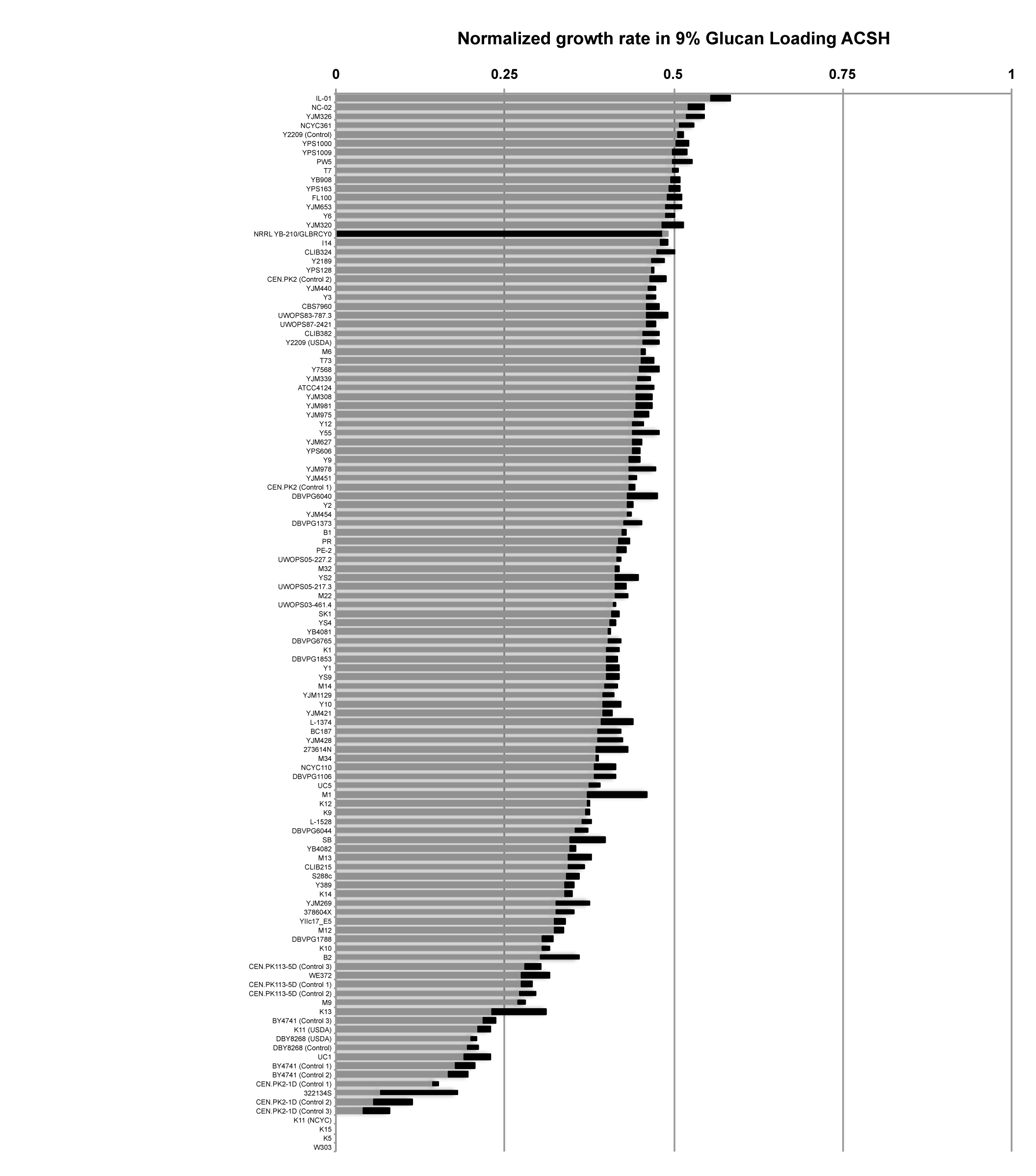

Supplement: Figure S3 — Bar graph displaying average growth rates (grey bars) of wild and domesticated S. cerevisiae strains in 9% glucan loading ACSH relative to YPD. Averages and standard deviations (black bars) are calculated from at least biological replicates. The row location for NRRL YB-210/GLBRCY0 strain used in this study is identified by opposite coloration (average growth rate in black, standard deviation in grey). (TIF) [file pone.0107499.s003.tif]

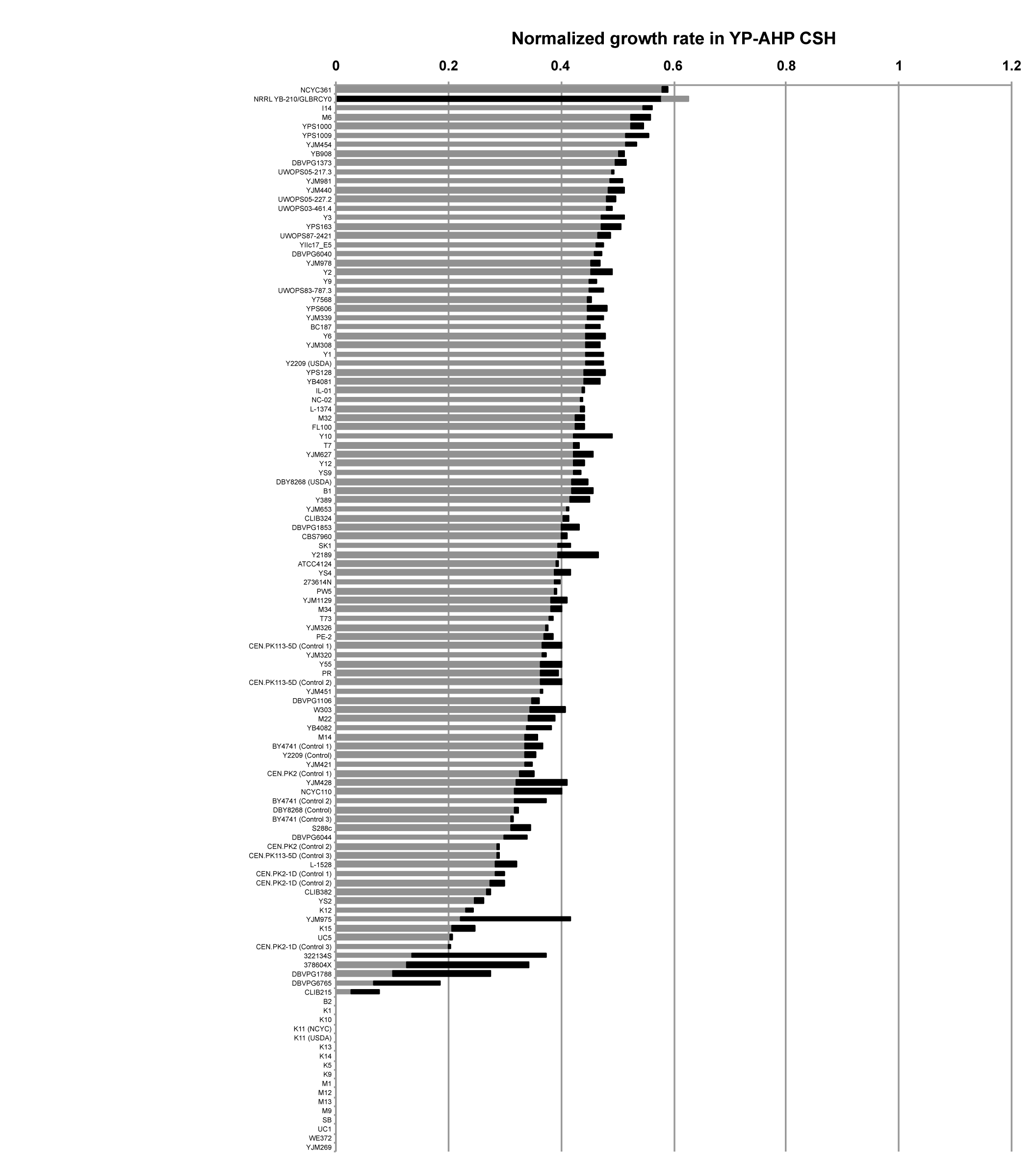

Supplement: Figure S4 — Bar graph displaying average growth rates (grey bars) of wild and domesticated S. cerevisiae strains in YP-AHP CSH relative to YPD. Averages and standard deviations (black bars) are calculated from at least biological replicates. The row location for NRRL YB-210/GLBRCY0 strain used in this study is identified by opposite coloration (average growth rate in black, standard deviation in grey). (TIF) [file pone.0107499.s004.tif]

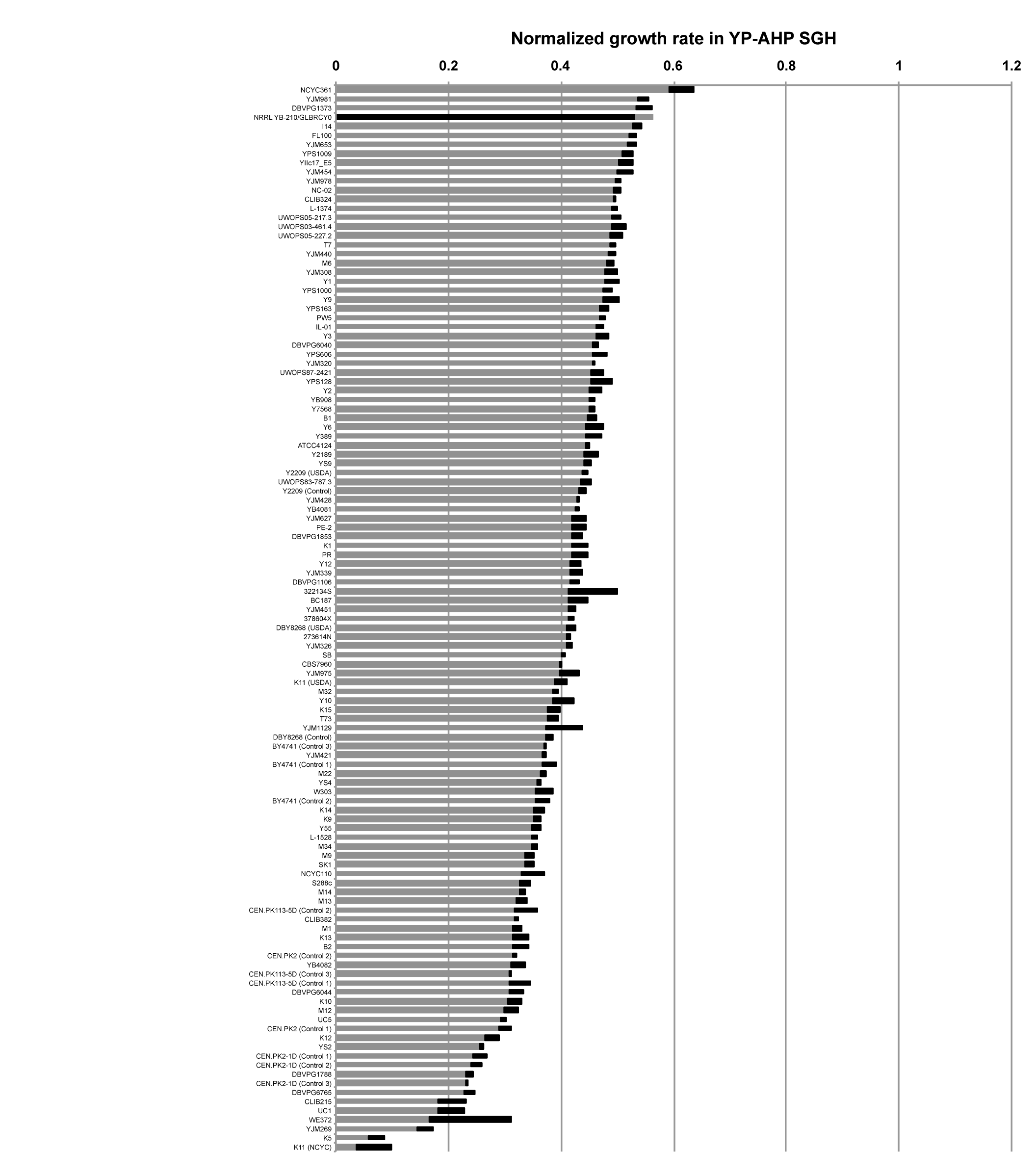

Supplement: Figure S5 — Bar graph displaying average growth rates (grey bars) of wild and domesticated S. cerevisiae strains in YP-AHP SGH relative to YPD. Averages and standard deviations (black bars) are calculated from at least biological replicates. The row location for NRRL YB-210/GLBRCY0 strain used in this study is identified by opposite coloration (average growth rate in black, standard deviation in grey). (TIF) [file pone.0107499.s005.tif]

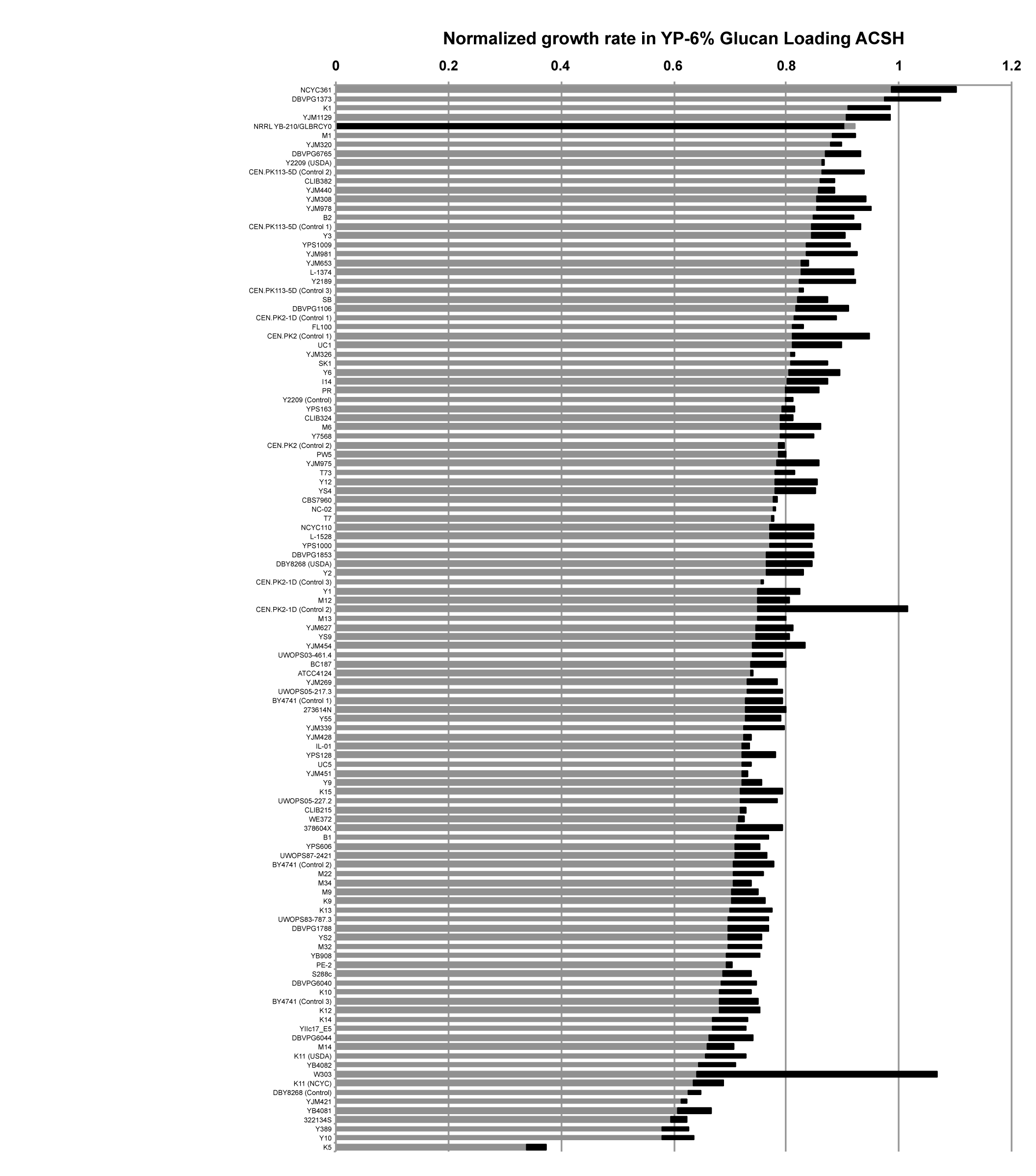

Supplement: Figure S6 — Bar graph displaying average growth rates (grey bars) of wild and domesticated S. cerevisiae strains in YP-6% glucan loading ACSH relative to YPD. Averages and standard deviations (black bars) are calculated from at least biological replicates. The row location for NRRL YB-210/GLBRCY0 strain used in this study is identified by opposite coloration (average growth rate in black, standard deviation in grey). (TIF) [file pone.0107499.s006.tif]

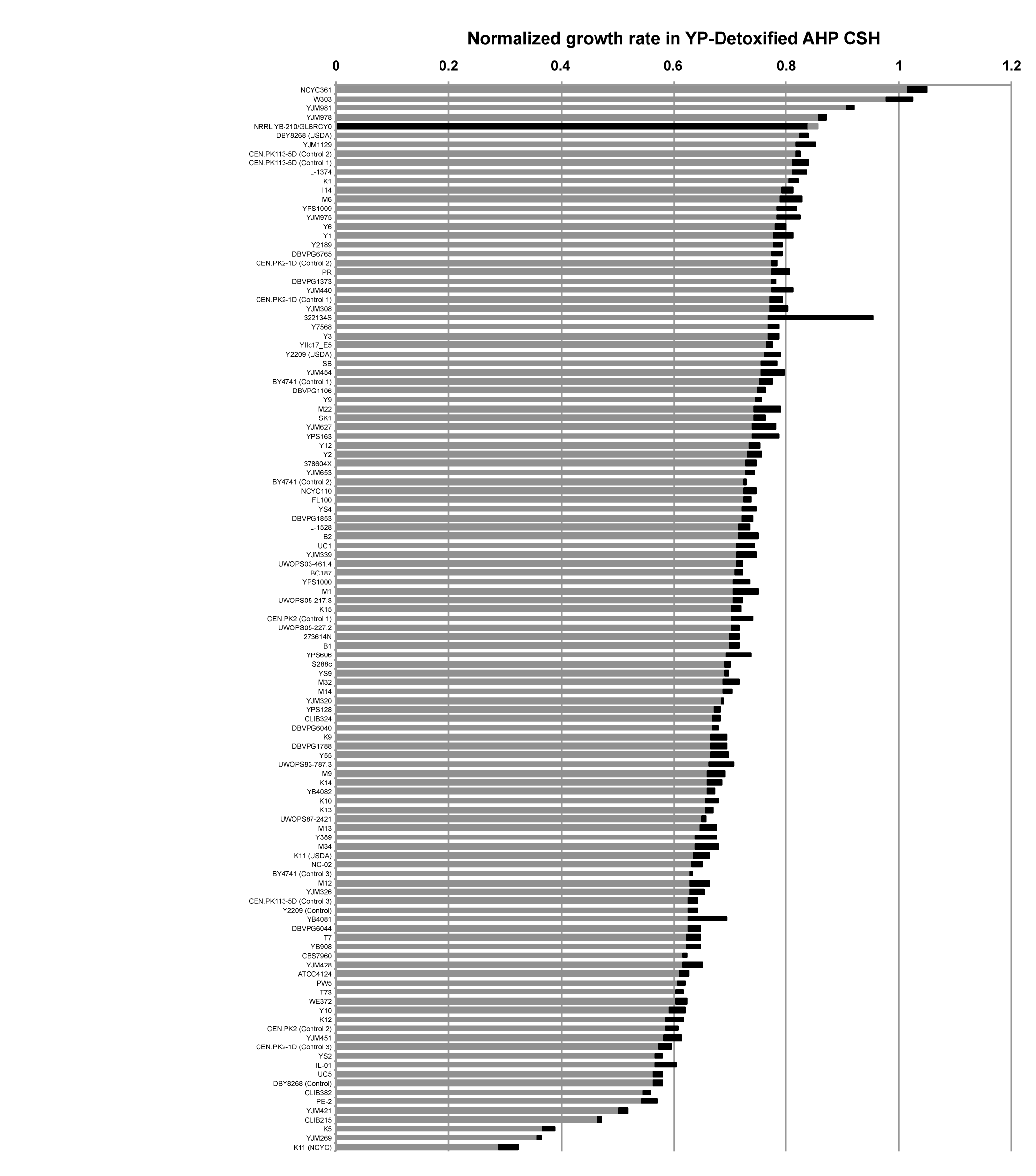

Supplement: Figure S7 — Bar graph displaying average growth rates (grey bars) of wild and domesticated S. cerevisiae strains in YP-detoxified AHP CSH relative to YPD. Averages and standard deviations (black bars) are calculated from at least biological replicates. The row location for NRRL YB-210/GLBRCY0 strain used in this study is identified by opposite coloration (average growth rate in black, standard deviation in grey). (TIF) [file pone.0107499.s007.tif]

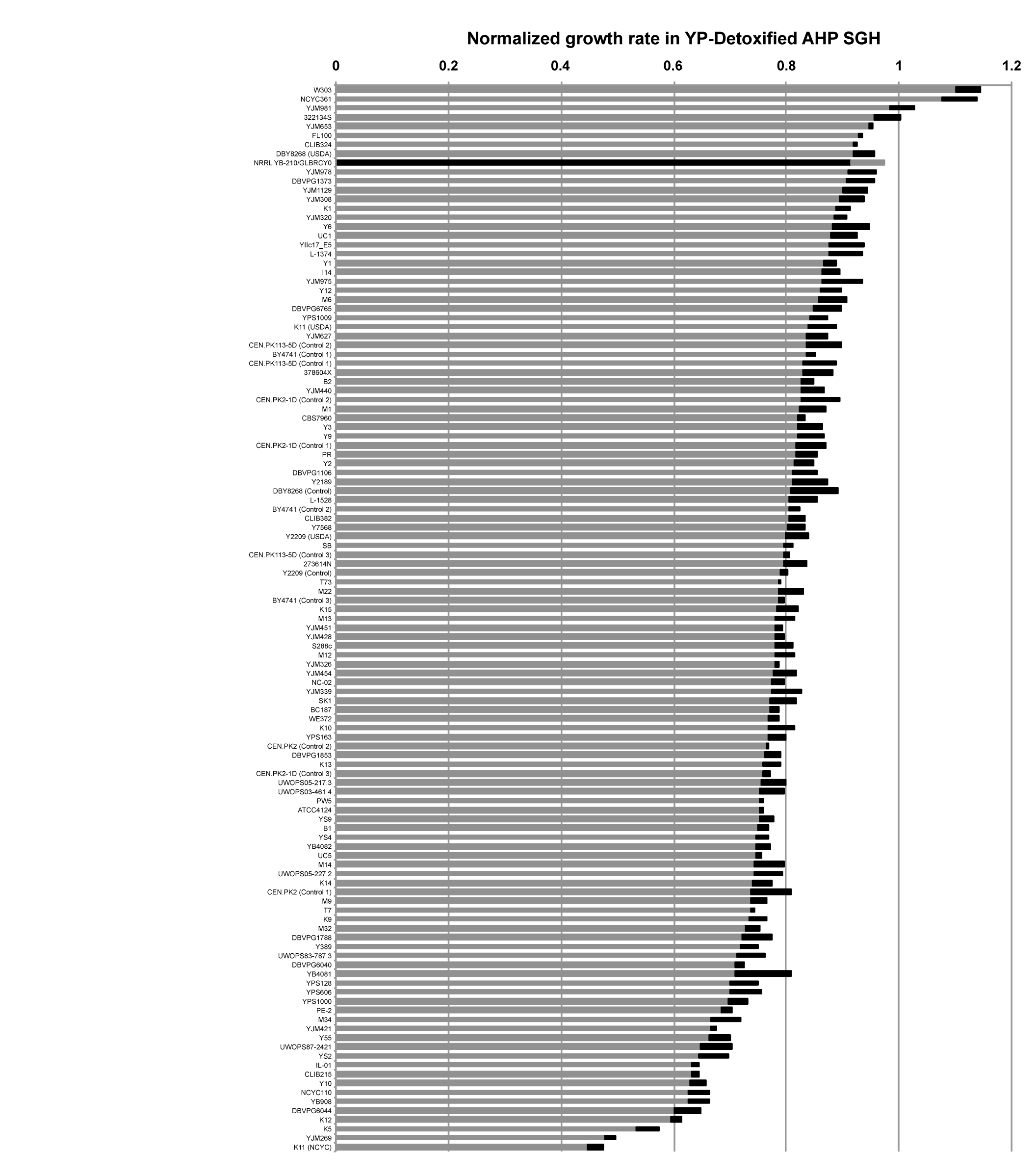

Supplement: Figure S8 — Bar graph displaying average growth rates (grey bars) of wild and domesticated S. cerevisiae strains in YP-detoxified AHP SGH relative to YPD. Averages and standard deviations (black bars) are calculated from at least biological replicates. The row location for NRRL YB-210/GLBRCY0 strain used in this study is identified by opposite coloration (average growth rate in black, standard deviation in grey). (TIF) [file pone.0107499.s008.tif]

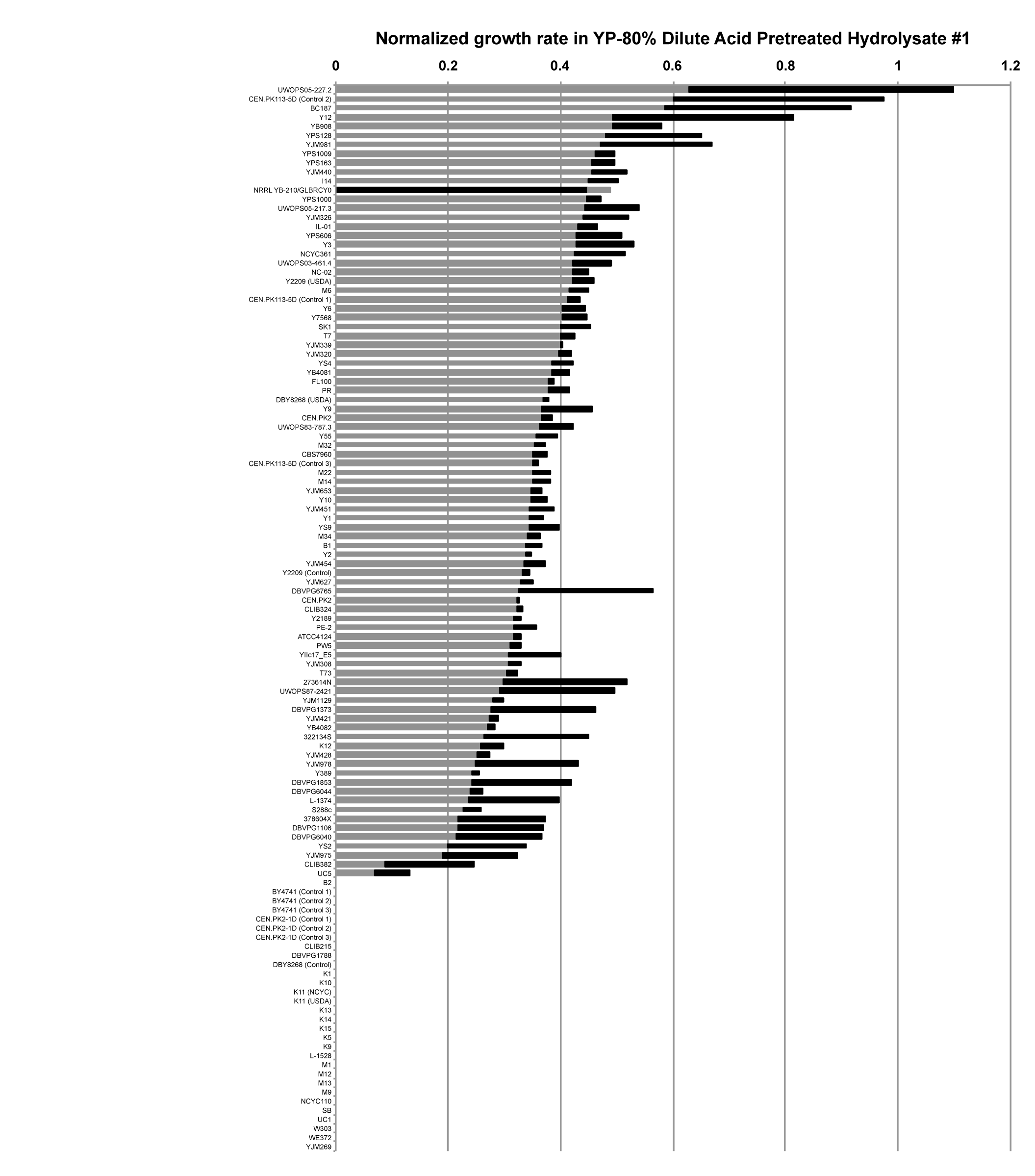

Supplement: Figure S9 — Bar graph displaying average growth rates (grey bars) of wild and domesticated S. cerevisiae strains in YP-80% dilute acid pretreated hydrolysate #1 relative to YPD. Averages and standard deviations (black bars) are calculated from at least biological replicates. The row location for NRRL YB-210/GLBRCY0 strain used in this study is identified by opposite coloration (average growth rate in black, standard deviation in grey). (TIF) [file pone.0107499.s009.tif]

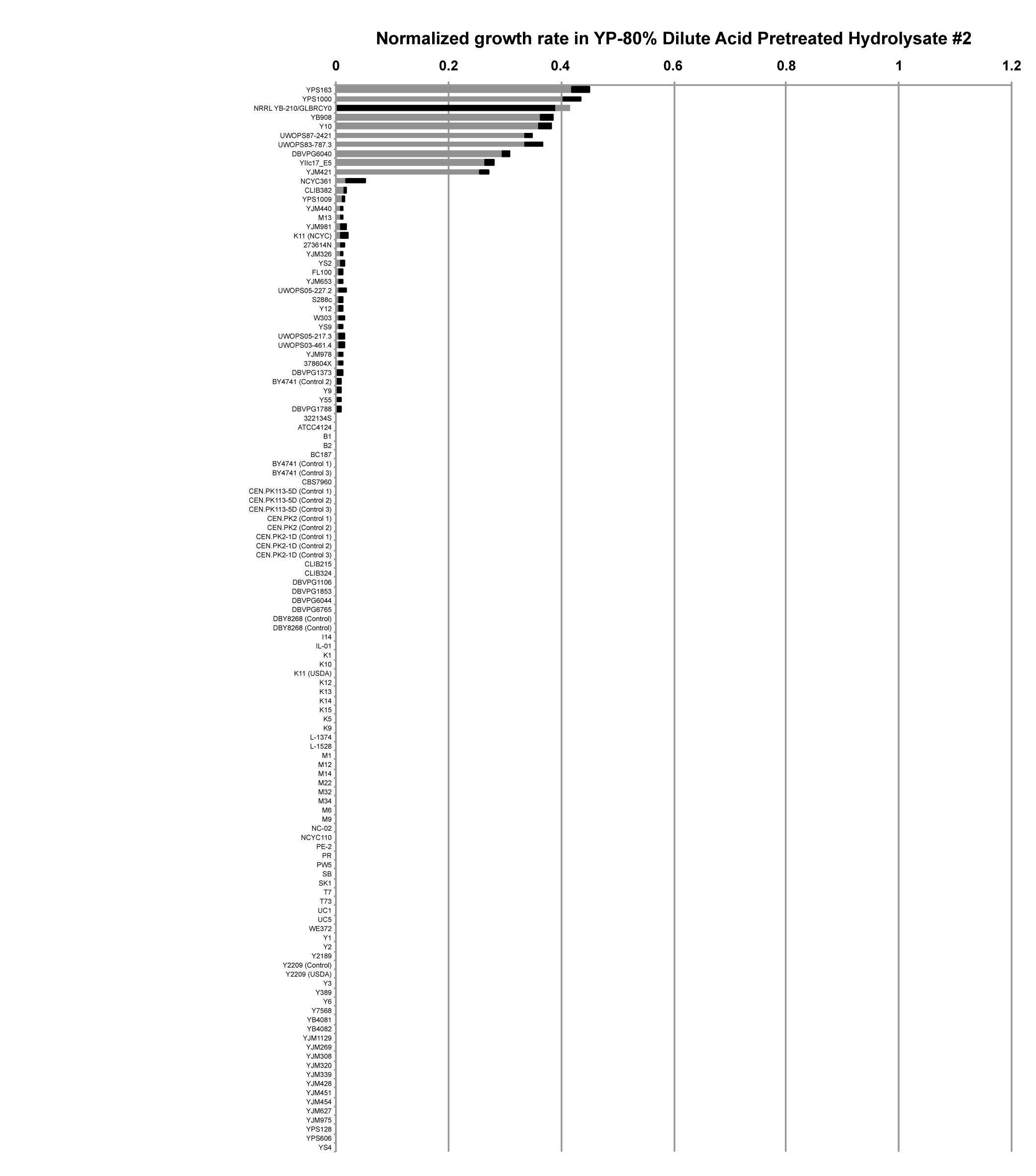

Supplement: Figure S10 — Bar graph displaying average growth rates (grey bars) of wild and domesticated S. cerevisiae strains in YP-80% dilute acid pretreated hydrolysate #2 relative to YPD. Averages and standard deviations (black bars) are calculated from at least biological replicates. The row location for NRRL YB-210/GLBRCY0 strain used in this study is identified by opposite coloration (average growth rate in black, standard deviation in grey). (TIF) [file pone.0107499.s010.tif]

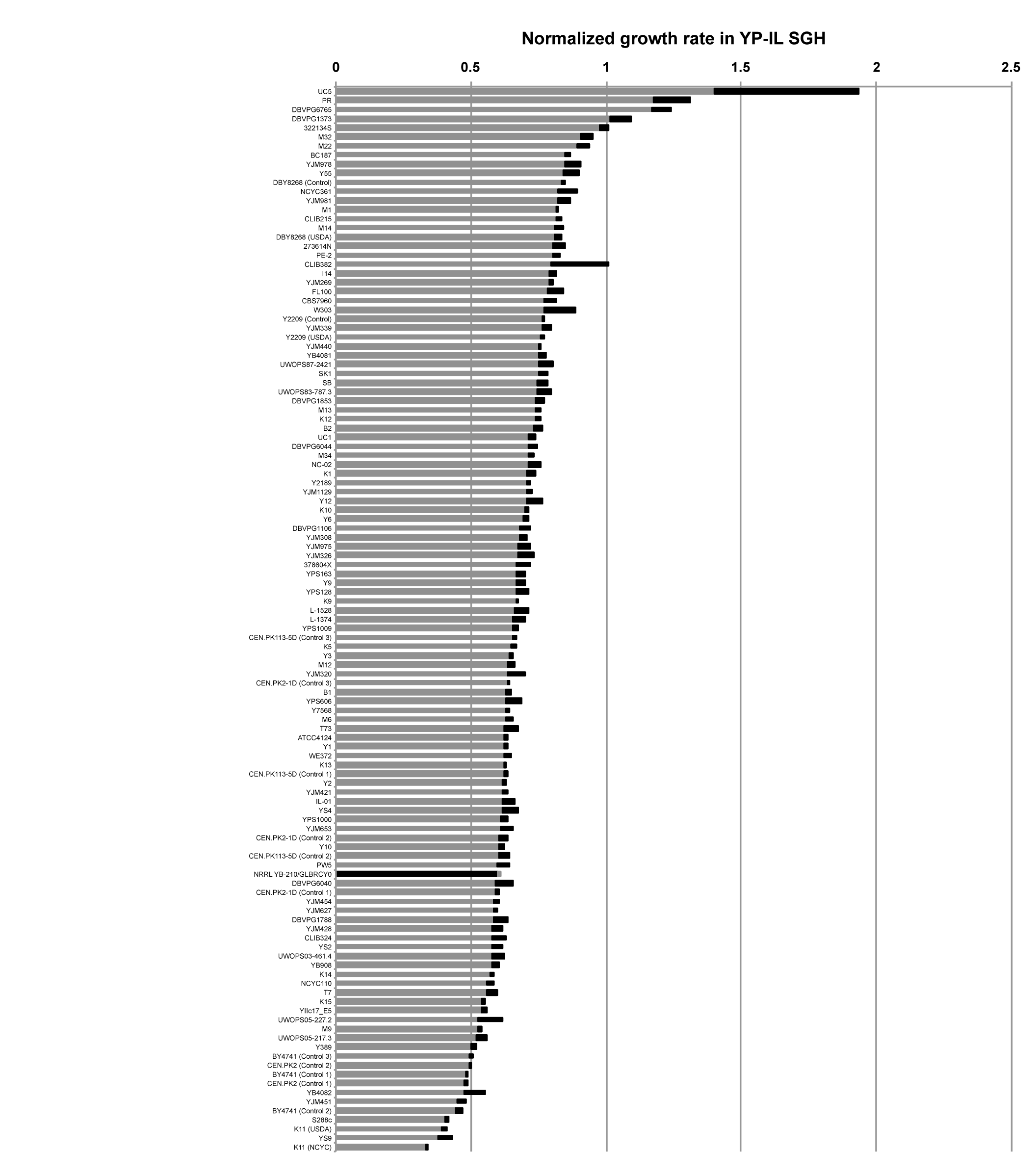

Supplement: Figure S11 — Bar graph displaying average growth rates (grey bars) of wild and domesticated S. cerevisiae strains in YP-IL SGH relative to YPD. Averages and standard deviations (black bars) are calculated from at least biological replicates. The row location for NRRL YB-210/GLBRCY0 strain used in this study is identified by opposite coloration (average growth rate in black, standard deviation in grey). (TIF) [file pone.0107499.s011.tif]

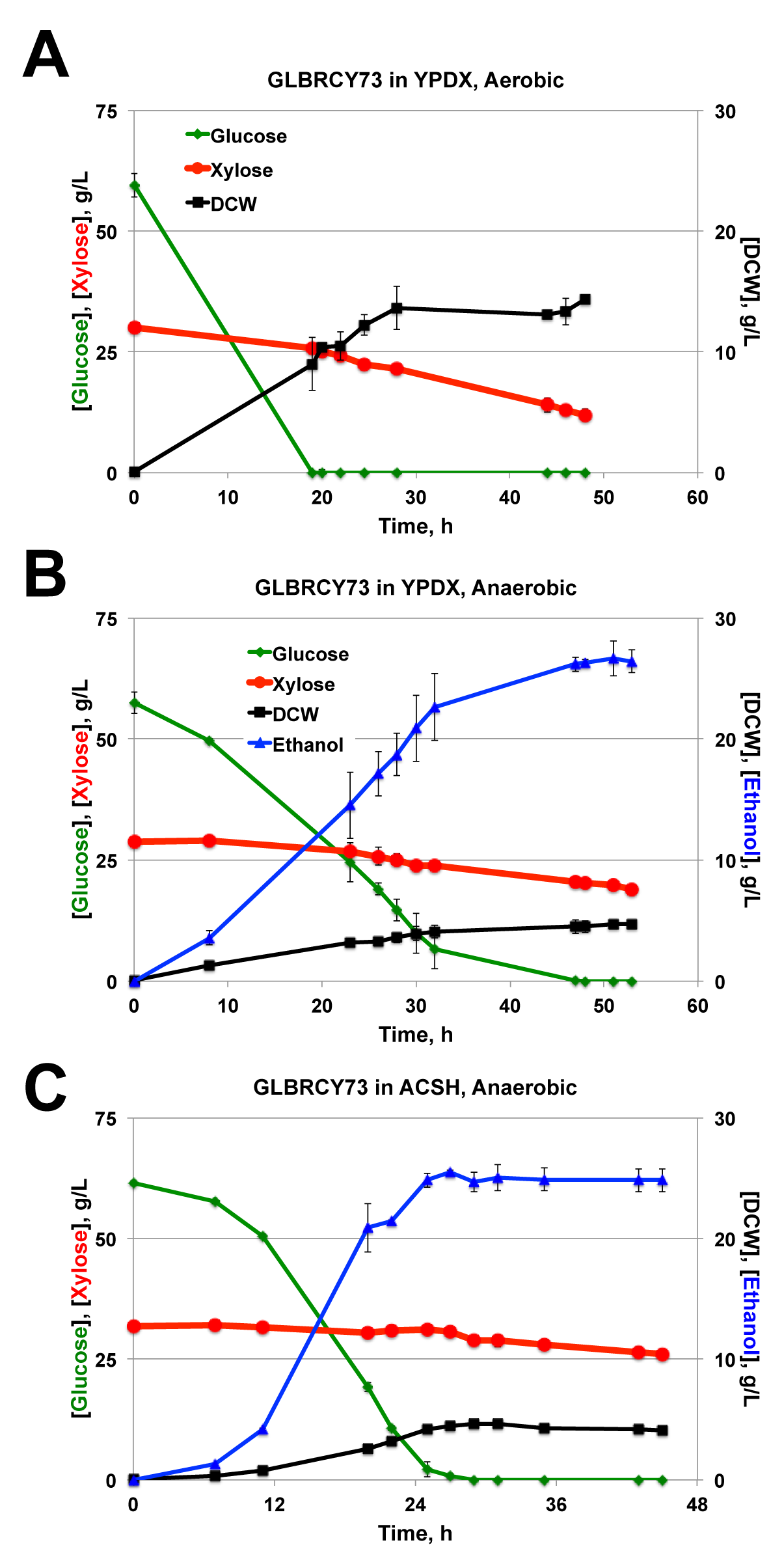

Supplement: Figure S12 — Hydrolysate-tolerant YB-210/GLBRCY0 engineered with XR/XDH and evolved for aerobic xylose metabolism does not ferment xylose anaerobically. The YB-210/Y0 strain engineered with XYL1, 2 and 3 genes from S. stipitis and aerobically-evolved (GLBRCY73) was cultured in bioreactors and evaluated for consumption of xylose in aerobic YPDX (A), anaerobic YPDX (B) and anaerobic ACSH (C) media as described in Materials and Methods. Concentrations (g/L) of glucose (green), xylose (red), dry cell weight (black) and ethanol (blue) are averages and standard deviations from two independent biological replicates. (TIF) [file pone.0107499.s012.tif]

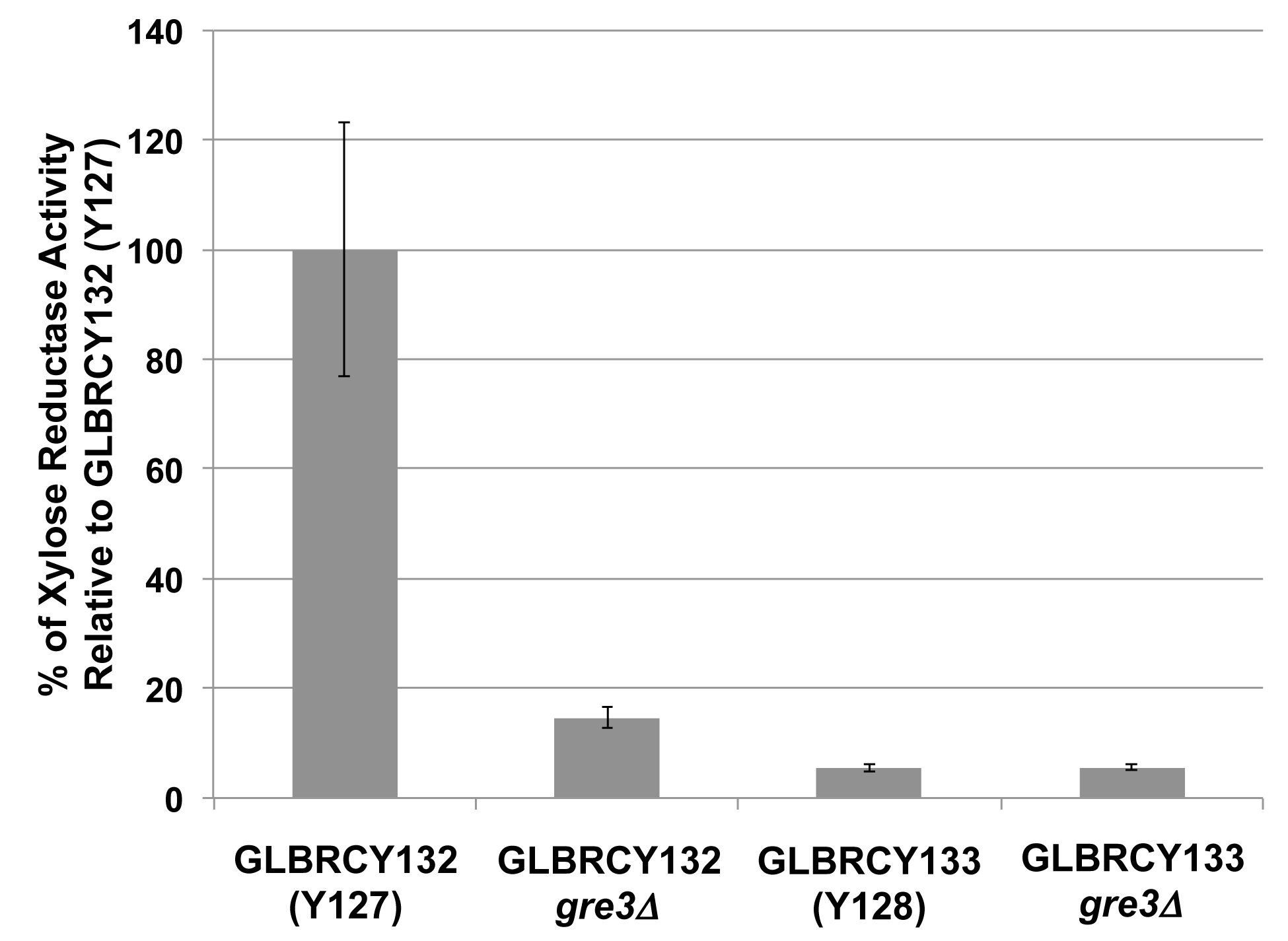

Supplement: Figure S13 — GLBRCY133 (Y128) cell extracts display reduced in vitro xylose reductase activity similar to GRE3 deletion strains. The indicated strains were cultured aerobically in YPD, harvested and prepared for in vitro xylose reductase activity assays as described in Materials and Methods. Xylose and NADPH were added to each extract, and then rates of change in absorbance at 340 nm were measured to determine the Units of enzymatic activity normalized to mg of total protein in the cellular extract. The graph displays the average percent of in vitro xylose reductase activities and standard deviations of indicated strains relative to GLBRCY132 (marker rescued GLBRCY127, which contains wild-type GRE3) in biological duplicate. (TIF) [file pone.0107499.s013.tif]
